# Role of the Copper Microstructure on Ethylene Stability during CO_2_ Electrolysis

**DOI:** 10.1021/acsenergylett.6c00513

**Published:** 2026-03-11

**Authors:** Jesse Kok, Nikita Kolobov, Mohammed Sharah, Amirhossein Foroozan, Shayan Angizi, Konstantinos Dimitriou, Drew Higgins, Thomas Burdyny

**Affiliations:** † Department of Chemical Engineering, 2860 Delft University of Technology, van der Maasweg 9, 2629 HZ Delft, The Netherlands; ‡ e-Refinery Institute, 2860 Delft University of Technology, Leeghwaterstraat 39, 2628 CB Delft, The Netherlands; § Department of Chemical Engineering, 3710 McMaster University, Hamilton, Ontario L8S 4L7, Canada

## Abstract

Catalyst lifetime is a primary technical bottleneck obstructing Cu-based CO_2_ reduction (CO_2_R), with restructuring via dissolution-redeposition being a commonly reported reason for selectivity loss. Here we examine how atomistic restructuring manifests at the microlevel of gas diffusion electrode (GDE)-based systems, ultimately compromising long-term CO_2_R performance. Using a flow-cell CO_2_R electrolyzer configuration and a copper-coated PTFE GDE, we first show how voltage gradients result in directional in-plane copper migration and porosity changes, causing a decrease in CO and ethylene production due to blocked catalyst pores. By the incorporation of different ionomer and inert carbon overlayers onto copper, we then demonstrate how in-plane degradation is mitigated by modulating the local pH and voltage homogeneity of the electrode, extending ethylene lifetimes by 10-fold. Ultimately, through-plane compaction of copper then becomes the limiting degradation pathway. Combined, these results provide rationale for the paradox of why copper degradation in membrane-electrode assemblies illustrates 100-fold greater stabilities than H-cell and flow-cell architecture.

## Graphical abstract

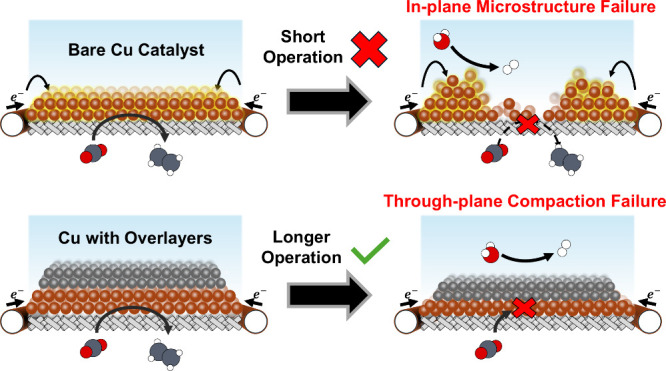

The electrochemical conversion of carbon dioxide (CO_2_) into multicarbon products (C_2+_-products) on copper (Cu) electrodes has progressed extensively,^1–3^ with combined theoretical and applied innovations advancing performance benchmarks.^4^ The utilization of porous gas diffusion electrodes (GDE), which enable short diffusion distances for CO_2_ vapor into porous catalyst layers,^5–7^ has been a particularly key enabler of increasing reaction current densities.^8–11^ Yet, state-of-the-art CO_2_ electrolyzers fall short of achieving competitive energy efficiencies^12,13^ and system lifetimes, with the latter being the most substantial barrier to overcome.^14,15^

Pseudostable operation of 40,000–60,000 h is required to minimize the capital expenditures of CO_2_ electrolyzers,^14–16^ yet most works utilizing industrially relevant conditions struggle to reach 100 h of operation.^15,17^ Specifically, within tens of hours, most copper-based CO_2_ electrolyzers demonstrate a shift in selectivity from predominantly C_2+_ products to producing almost entirely hydrogen via the competing hydrogen evolution reaction (HER).^18^ While destabilization of the electrode performance can be a result of salt formation,^19–22^ flooding,^23–25^ or impurity deposition,^26,27^ once all of these factors are mitigated,^19,20,28^ selectivity losses over extended periods of operation are typically ascribed to the rapid restructuring of copper catalysts.^29–34^

The tendency of copper to restructure emanates from its low cohesive energy and high adsorption affinity toward oxygen-containing species.^22,35,36^ Notably, copper restructuring occurs both before and during electrochemical CO_2_ reduction (CO_2_R). Before electrolysis, copper experiences anodic dissolution at open-circuit potential.^33,37–39^ Upon the application of an adequately reducing potential (<0 V vs RHE), the dissolved copper ions then redeposit onto the electrode.^29,33^ During CO_2_R, substantial morphological changes also take place over short time scales.^29,33,40^ Recently, restructuring was shown to occur through copper–carbonyl complexes (CuCO)^+^ dissolving from the surface of the electrode, dissociating into Cu^+^ and CO, and then Cu^+^ redepositing back onto the copper surface.^31^ In another study, dissolved copper species were found to migrate from the catalyst layer into the supporting carbon gas diffusion layer (GDL).^41^ The surface-mobility of copper due to the presence of *CO adsorbates (where * indicates a species adsorbed on the catalyst surface) has also been described by density functional theory (DFT) and examined by inductively coupled plasma mass spectroscopy (ICP-MS), *in situ* liquid-cell transmission electron microscopy (TEM), and *in situ* Raman spectroscopy.^32,42–45^ Most importantly, restructuring is heavily correlated to CO_2_R mechanisms itself, meaning that as long as CO_2_R occurs, copper will persist through continuous dissolution-redeposition cycles.^46–49^

While the cause and observation of copper restructuring due to CO_2_R are well-supported, the link between restructuring and changes in selectivity is indirect. Most previous works have hypothesized that restructuring leads to changes in the exposed copper facets over time, and these changes subsequently lead to a loss of CO_2_R selectivity.^48,50–53^ Several atomistic approaches such as carbon coatings^9,54^ and metallic oxide coatings^55,56^ have subsequently been proposed to mitigate copper restructuring to improve electrode stability. An overlooked aspect of copper restructuring, however, is how morphological changes impact the CO_2_R activity at the microscale. For high current density GDE-systems that depend on a porous catalyst layer for CO_2_ access, restructuring greatly changes the porosity and distribution of copper over time. To date, these destabilizing effects of the microstructure have yet to be experimentally examined as a driver for the loss of CO and ethylene selectivity over time.

Within this work, we wanted to examine how atomistic restructuring drives changes in the microscale structure of a copper catalyst on a GDL and how such changes may contribute to CO_2_R destabilization over time. Beginning with a copper catalyst sputtered on a nonconductive polytetrafluorethylene (PTFE) GDE, we show using *ex situ* scanning electron microscopy (SEM) and inductively coupled plasma optical emission spectroscopy (ICP-OES) that nonuniform potential gradients on the electrode lead to a 10% directional in-plane migration of copper mass after 1 h of operation. These changes correlate to reduced CO_2_R selectivity and imply that, under severe restructuring of the copper catalyst, CO_2_ access from the gas-phase may be inhibited. By increasing the local pH with an ionomer coating or improving voltage homogeneity with carbon overlayers on the copper catalyst layer, these in-plane copper migration effects are shown to be reduced, allowing for CO_2_R lifetimes to be extended up to 10-fold. The results show that restructuring has a preferential direction along potential gradients, implying that any in-plane or through-plane potential gradients will influence the microstructure of copper over time. These findings are then put into the context of different CO_2_R cell architectures that show varying levels of resistance against microstructure changes.

In a previous work analyzing pulsed CO_2_ electrolysis on a PTFE GDL, we demonstrated through top view SEM imaging that copper at the center of the GDE was nearly depleted after 20 h of electrolysis, while copper accumulated near the current collector.^57^ We hypothesize this copper directionality toward the current collectors was driven by the low in-plane conductivity of the copper-PTFE architecture, which leads to high voltage gradients. We then wanted to understand if such directional migration was occurring under continuous operation, and how copper migration and microstructure correlates to time-resolved changes in CO_2_R selectivity.

For the experiments, we utilized a PTFE-supported 500 nm sputtered copper catalyst layer (Figure S1). CO_2_ was fed in the vapor phase, while the flowing catholyte was 1 M KHCO_3_. In this setup, salt precipitation and flooding are reduced.^58^ Flow-cell systems also allow for easier post-mortem analysis of the catalyst layer, as there is no compression against an ion-exchange membrane. Notably, a neutral pH operating condition using KHCO_3_ gives much lower copper stabilities than KOH-fed systems, but KOH is not a viable electrolyte due to parasitic interactions with CO_2_.

We then subjected the electrode to a current density of −200 mA·cm^–2^ inside a polytetheretherketone (PEEK) flow cell for 100 min, during which the gas products were analyzed using gas chromatography (see Materials and Methods for details). A schematic overview of the setup can be found in Figure S2–S3. The cathode potential as a function of time is shown in Figure S4, with minimal changes in the *iR*-uncorrected electrode potential observed during operation. As is characteristic in H-cell and flow-cell literature, the gaseous Faradaic efficiencies (FE) for CO_2_R shifts from predominantly ethylene to entirely the hydrogen evolution reaction within 100 min (Figure 1a). Here CO experiences a steady degradation from the start of the experiment, while ethylene production drops sharply after 1 h. Ethylene decreases correlate with a temporary increase in methane and a steady increase in hydrogen. The consistent operating time and decay rate of ethylene in the quadruplicate experiments highlights the replicability of the failure mechanism (see Figure S5 for the FE profiles of independent experiments). From here onward, we define a >5% decrease in ethylene FE as the failure time for the system. For two test cases, liquid products were also characterized at 30 and 80 min (Figure S6), indicating that product quantification nears 100% FE.

**Figure 1:**
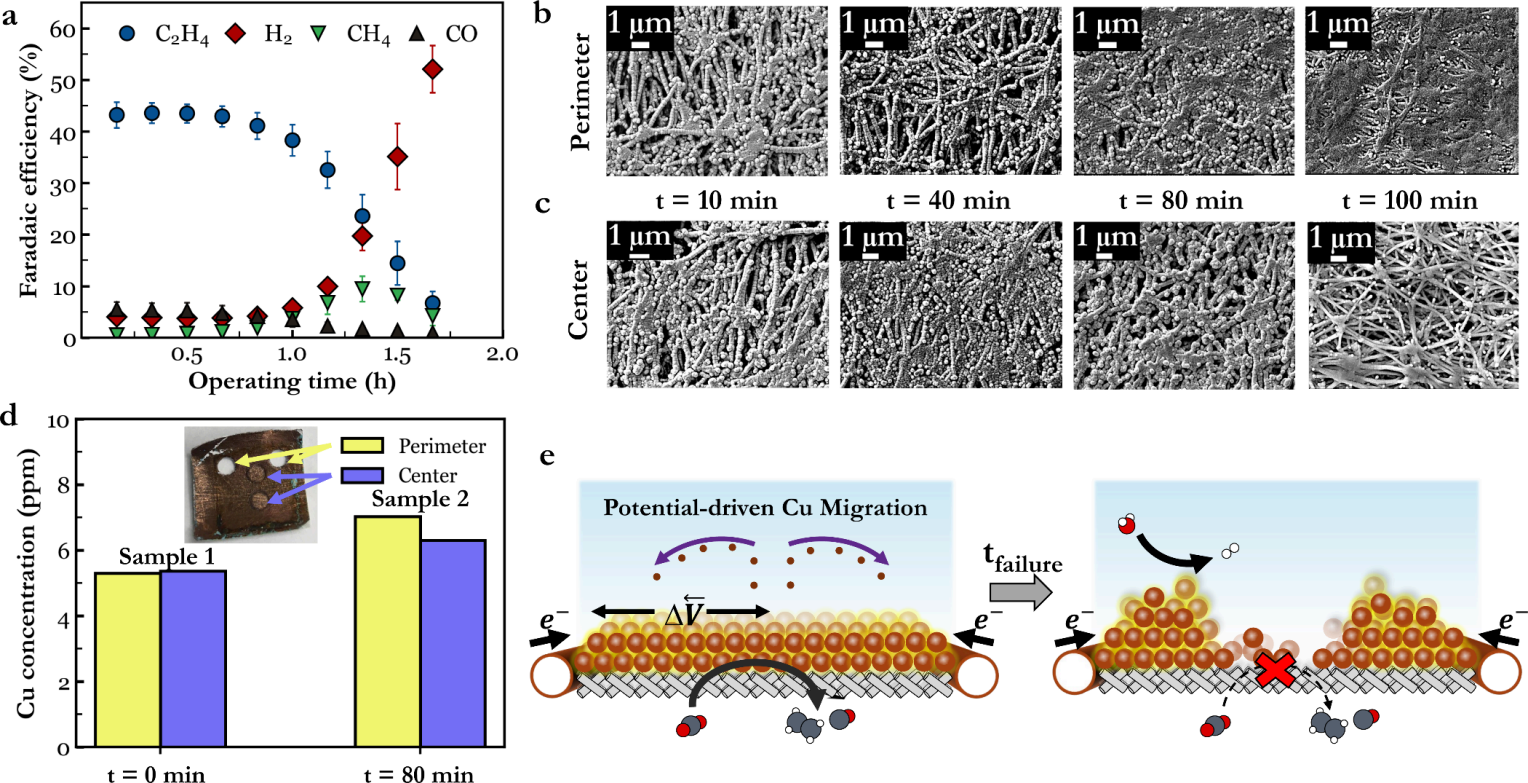
(a) Faradaic efficiency profiles of gas products during CO_2_ electroreduction on the copper GDE at −200 mA·cm^–2^. Error bars represent the standard deviation of at least three separate experiments. SEM imaging on the perimeter (b) and center (c) of copper GDEs after CO_2_ electrolysis at −200 mA·cm^–2^ for different operational times. The indicated times represent the amount of electrolysis time before the samples were removed for *ex situ* SEM analysis. (d) ICP-OES data with the average copper mass loading across the perimeter and center for two different samples, a pristine copper GDE without electrolysis, and a copper GDE after 80 min of operation. The inset shows the 3 mm punctures used for the *t* = 80 min analysis. The direct center was used for the FIB analysis and thus not removed. (e) A schematic representation of potential gradient (Δ V⃗ )-driven substantial in-plane migration of copper due to dissolution-reposition restructuring.

We then compared the capacitance of the pristine and used cathodes (after 80 min of operation) observing a 25% drop in double layer capacitance (see SI, Figure S7, and Table S1). These decreases indicate a loss of electrochemically active surface area (ECSA), which could be a result of catalyst agglomeration and porosity decreases. On a carbon GDL, an increase in ECSA is commonly observed when flooding occurs,^59,60^ but this is not the case for the PTFE GDE. Impurity deposition is neglected as the cause of FE degradation as the ratio of the catholyte volume to the geometric surface area of the cathode is rather low.^61^ Lastly, no salt particles were observed after operation (Figure S8).

With the baseline set, we ran individual experiments in which electrolysis was stopped at preset times corresponding to different ethylene selectivity regimes. These time delineations denoted numerically in Figure 1a-b represent (1) a sample at peak ethylene selectivity (10 min), (2) a sample demonstrating minimal selectivity loss (40 min), (3) a sample experiencing ∼50% ethylene selectivity loss (80 min), and (4) a 90% ethylene selectivity loss (100 min). The FE profiles of each test are shown in Figure S9. For each case, both the perimeter (Figure 1b) and center (Figure 1c) of the 1.5 cm × 1.5 cm PTFE-based GDEs were examined using *ex situ* SEM analysis. Up to 40 min of operation no clear differences are observed in the copper microstructure between the center and perimeter. At 80 and 100 min, however, morphology differences become clearer, with porosity increasing the electrode center and decreasing at the perimeter. Specifically, after 100 min of operation, much of the originally deposited copper particles at the center are no longer present on the PTFE fibers (Figure 1c), while larger copper islands have accumulated at the perimeter (Figure 1b and Figure S10–S11). In the perimeter regions, copper closer to the vapor phase then loses access to electrolyte and ionic pathways, while copper islands exposed to the electrolyte have little access to CO_2_.

To characterize changes to the microstructure further, we compared the cross sections of a pristine and 80 min sample prepared by Focused Ion Beam (FIB) microscopy and the spatially resolved quantities of copper using Inductively Coupled Plasma Optical Emission Spectroscopy (ICP-OES). For the ICP-OES analysis, two circular (3 mm diameter) samples were taken out of both the center and perimeter of the GDE (Figure 1d). For the 80 min sample, a 10% higher copper signal was seen in the perimeter compared to the center, indicating a net migration of copper as ethylene selectivity decreases (Figure 1d, Table S2). The FIB cross-section analysis could not be used for porosity analysis due to the high variability of the PTFE support, but cross-sectional images highlight copper deposition onto both the upper and lower PTFE fibers (Figure S12). More conformal copper coating is also present in PTFE nodes common for expanded PTFE (Figure S1a). It is unclear if the lower fiber copper is conductively connected to the current collector. Throughout operation, any copper areas that are, or become, electrically disconnected are subject to anodic dissolution and subsequent redeposition onto copper surfaces which are under a reducing potential.^35,37^

Combined, the above characterizations highlight a directional migration of copper toward the current collector. From the physical characterization of the fully failed sample (100 min), we conclude that a loss of CO_2_R selectivity is occurring due to both depletion of copper in the center of the electrode and accumulation of copper in the perimeter regions. In the case of the accumulated regions at the electrode perimeter, excess copper can block CO_2_ vapor access (Figure 1e), leading to HER when under fixed current density operation. A temporary increase in methane FE is also observed as CO becomes almost fully depleted and ethylene selectivity begins dropping sharply. As methane also originates from *CO intermediates,^62^ these observations could indicate that *CO coverage is insufficient for intermediate coupling toward ethylene production.^1,63^ Conversely, while the center regions of the fully failed sample may still contain copper with CO_2_ access, the decreasing catalyst layer conductivity increases the in-plane potential gradients further. Disproportionately more current is then forced to the perimeter regions, as we previously showed when a 50 nm thick copper layer was examined with infrared thermography.^64^ Thus, a negative feedback mechanism exists where regions with ample CO_2_ access have continuously lower potentials and subsequently contribute less to the total current. As restructuring is also linked to the CO_2_R mechanism itself, regions with greater CO_2_R experience higher material migration. These overlapping effects support the observed sharp drop in ethylene FE over time as compared to more gradual changes.

We hypothesize that the directional driving force for copper migration is a result of in-plane potential gradients, which influences copper deposition current.^64,65^ Thus, positively charged copper species in the electrolyte will preferentially electrodeposit on the more negative potential regions closer to the current collectors.^33,44,66^ While the individual migration distance of a singular copper species may be relatively small, continuous repetition of this phenomenon leads to a severe morphology change. Critically, we believe the change in microstructure to be the dominant CO_2_R degradation mechanism on these time scales. More importantly, while our observations are on a nonconductive PTFE GDL, the implications are that any form of potential gradients will inevitably cause a directional migration of dissolved copper species over long enough times, even on a conductive carbon GDL.^65^ For example, the migration of copper from a catalyst layer into a flooded carbon GDL was also observed.^41^

To further examine the effects of potential gradients, we performed an experiment where the current collector was applied only to the upper half of the electrode and not fully around the perimeter (Figure S13a). The electrode is then exposed to much greater potential gradients as the maximum distance to the current collector increases from 0.75 to 1.5 cm (Figure S13b, Figure S14). The FE profiles of carbon monoxide and ethylene at −200 mA·cm^–2^ subsequently experienced much faster declines in ethylene production, despite beginning at the same value (Figure S13c). Again, we also see a temporary increase in methane FE, which is now shifted to early times (Figure S13d). An *ex situ* SEM analysis again shows a clear migration of the copper catalyst toward the current collector after 1 h, with the formation of a compact copper layer (Figure S15). These results again correlate microstructure changes with losses in selectivity.

With the demonstrated link between potential-driven microscale restructuring and temporal changes in selectivity, we then anticipate that slowing copper restructuring should lead to more stable ethylene production. Restructuring itself can then be dissected into three mechanistic components: dissolution, migration and redeposition (Figure 2a). Eliminating the dissolution of Cu-complexes would help to circumvent restructuring altogether but is challenging from a materials-perspective without changing reactivity. Furthermore, since Cu-complex dissolution has been linked to Cu-CO formation, it is directly coupled to the CO_2_R current density. The link between dissolution rate and current density is a factor in why lower current density experiments show greater ethylene lifetimes.^65^ The two-remaining means of influencing restructuring are then through limiting migration of copper complexes and encouraging fast redeposition, both of which reduce the net distance traveled by a dissolved copper species after dissolution. By reducing the rate of restructuring, favorable copper microstructures can be then maintained over longer operating periods (Figure 2b).

**Figure 2:**
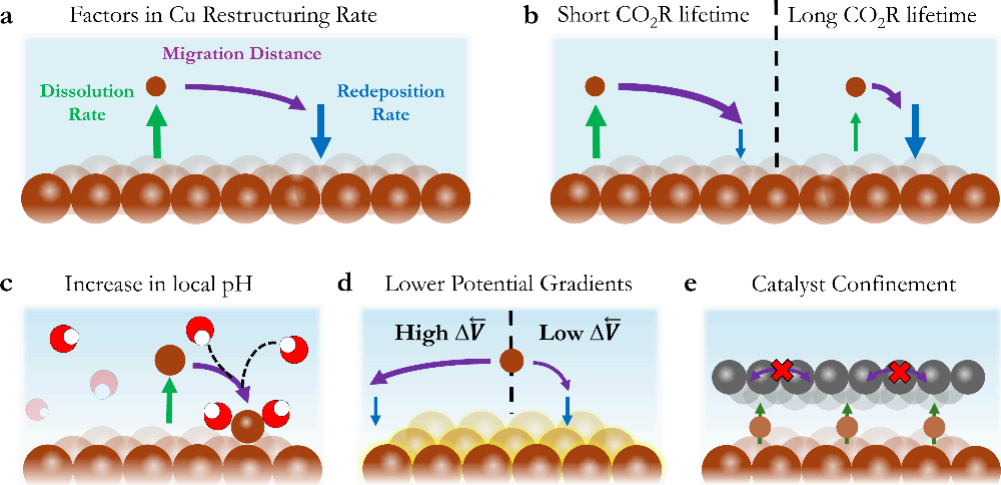
(a) Factors influencing the rate of copper restructuring including dissolution of Cu-complexes, migration, and redeposition. (b) Representative link between dissolution-migration-redeposition and CO_2_R lifetimes. Approaches that affect the distance traveled and/or dissolution rate of copper in favor of catalyst stability. (c) Illustration that higher local pH reduces catalyst layer restructuring. (d) How low vs high potential gradients across an electrode influence the migration distance of a dissolved Cu-complex. (e) Confinement of the copper catalyst layer by coating with an additional layer.

We then identify three generalized approaches to limit the distance traveled by Cu-species (Figure 2c-e). These approaches include (i) increasing the local alkalinity of the reaction environment, (ii) improving the potential distribution of the electrode (potential homogeneity), and (iii) physical confinement of the copper catalyst. These generalized approaches have been used in literature to extend CO_2_R lifetimes,^67^ either intentionally or unintentionally, yet their impact on stabilizing the microstructure of copper has yet to be specifically examined.

The use of locally alkaline environments has been shown to enable long-term CO_2_ electrolysis, with KOH-based electrolytes showing much greater stability than neutral-pH systems.^9,57,68^ Local alkalinity near the cathode is impacted by the electrolyte type, buffer capacity, applied current density, and catalyst/electrolyte mass transport. For example, in Figure S16–S17 we show that the use of 0.1 M KHCO_3_, which has a lower buffering capacity,^7^ results in longer stability compared to a 1 M KHCO_3_. The direct mechanism for greater copper stability at an elevated pH is unclear.

Limiting the migrative driving force is the next approach to slow potential-driven copper restructuring. Achieving a more uniform potential distribution across both the in-plane and through-plane direction of an electrode would decrease the directional migration of Cu, thus encouraging redeposition closer to the dissolution location (Figure 2d). Within an electrode, variations in the potential of the copper surface occur as a result of ohmic drops as electrons traverse from the current collectors across the electrode. Ohmic drop (Δ*V*) then scales linearly with the conductivity of the GDE and current density and roughly quadratically with the traversed distance from the current collector to a point on the electrode (Δ*V* ∝ *L* ^2^). Potential homogeneity across the electrode can then be encouraged by decreasing the spacing of current collectors or increasing the conductivity and cross-sectional area of the catalyst layer. Greater conductivity can be enabled through the use of carbon GDLs, thicker catalyst layers, and conductive overlayers. All porous conductive networks will suffer voltage drops, however, implying that some homogeneity will always be incurred in both the in-plane and through-plane directions.

Lastly, migration distances upon Cu dissolution and redeposition cycles can be reduced by spatially confining the active copper catalyst (Figure 2e). For example, depositing a non-CO_2_R active layer on top of a copper catalyst layer can inhibit the migration of the copper species to the bulk electrolyte. Furthermore, the introduction of ionomers in between copper particles limits copper species movement through a porous catalyst layer. Through both approaches, Cu-complexes become more likely to redeposit to where dissolution occurred, inhibiting the spatial variations in copper from Figure 1b-c.

To evaluate and disambiguate the effects of alkalinity, potential homogeneity, and confinement on CO_2_R stability, we compared catalyst layer architectures that originate from the initial 500 nm thick copper GDE (Figure 1). The modifications include the addition of cation (CEI) or anion (AEI) exchange ionomers and conductive-based coatings (carbon NPs) (see Methods). Figure 3a-c shows the FIB cross-sectional imaging of the deposited samples. Here the ionomer and carbon coatings are positioned on top of the copper and clearly distinguishable. All of the samples were then subjected to the same −200 mA·cm^–2^ electrochemical testing giving the FE resulting profiles in Figure 3d (see Figure S18 for potential profiles). All samples showed similar peak ethylene FE but with varied longevities. The use of conductive coatings did result in overall lower measured electrode potentials, supporting improved conductivity and lower ohmic drops across the catalyst layer. For the physical characterizations presented below, further characterizations were performed at set operating times to contrast with the bare copper case from Figure 1.

**Figure 3:**
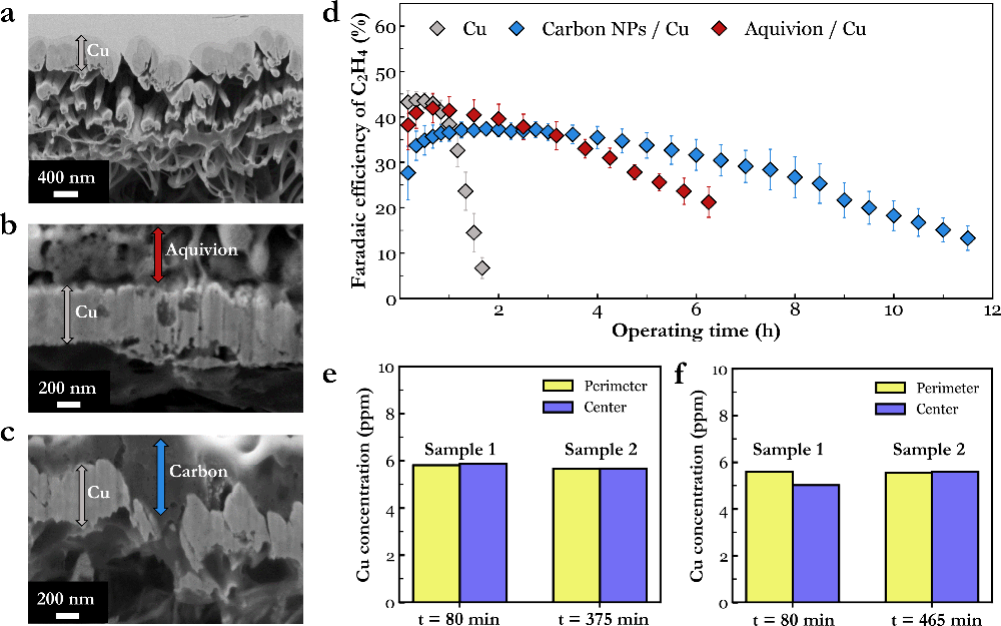
SEM images comparison of FIB cross-sections of the three electrode architectures used in the stability tests including (a) bare copper on PTFE, (b) copper coated with Aquivion, and (c) copper coated with Aquivion and carbon nanoparticles. All samples had a top-layer of tungsten applied for focused ion beam preparation. (d) Faradaic efficiency profile of ethylene as a function of operating time at −200 mA·cm^–2^ for the various copper electrodes. Error bars represent the standard deviation of at least three independent experiments. Independent experiment results with full gas product FEs are included in the SI. (e) ICP-OES data for perimeter and center segments of a two separate Aquivion/Cu electrodes taken after 80 and 375 min of operation. (f) ICP-OES data for perimeter and center segments of two separate carbon NPs/Cu samples taken after 80 and 465 min of operation.

Different ionomers act as a useful comparison case, as they provide similar confinement effects but different transport properties. Here a cation exchange ionomer (Aquivion) is compared against an anion exchange ionomer (Sustainion). For the case of an Aquivion ionomer overcoating, the loading was first optimized. While all loadings improved stability versus bare copper (Figure S19), an ionomer loading of 1.4 mg·cm^–2^ (9.2 mg/mL solution) extended ethylene production to >2 h of operation before a 5% decline in ethylene was observed. Moreover, the ethylene degradation rate slowed substantially versus the bare copper case. Conversely, the Sustainion ionomer case showed worse stability than the bare copper case for all loadings except 0.1 mg·cm^–2^ (Figure S20), which extended operation up to only 2 h.

Here we anticipate the disparity in stabilities of the ionomers to come from their effect on local electrode pH, which in turn slows copper restructuring. As a CEI, Aquivion lowers the transport of hydroxide and carbonate away from the surface of copper; the local pH is expected to be higher than the Sustainion case. Further, CEIs do not restrict potassium transport, which is beneficial for CO_2_ electroreduction (CO_2_ER).^63,69,70^ As copper shows much greater reported stability in KOH solutions, the more locally alkaline reaction environment created by CEIs mimics this local pH even in the neutral-pH KHCO_3_ solution. To examine the pH effect further, we performed CO_2_ electrolysis for the 1.4 mg·cm^–2^ Aquivion loading in both 0.1 M KHCO_3_ and 1 M KHCO_3_ catholyte solution. In contrast to the bare copper electrode where the electrolyte change did extend ethylene production by 2-fold (see Figure S17), switching to the 0.1 M KHCO_3_ electrolyte with Aquivion did not result in a noticeable change in stability (Figure S21). Also, the open-circuit potential (OCP) decay transient curves reveal a difference in local pH to arise when using none or different ionomers.^71^ Our previous work shows that the OCP curve generated after disconnecting the power supply following an ‘on’ period at a reductive current density is a function of the local hydroxide-ion concentration.^57^ The slower the OCP value transitions back to that of the pristine electrode in the electrolyte, the slower hydroxide-ions escape the local environment. The difference in pH between the bulk and the local environment are plotted for the bare Cu, Cu with Aquivion, and Cu with Sustainion as a function of time at OCP (Figure S22a). Aquivion-coated Cu experiences the slowest local pH change after operation, indicating successful trapping of hydroxide-ions. Furthermore, a small gradient in hydroxide ions is maintained with an Aquivion overlayer as compared to the other electrodes (Figure S22b).

To aim to characterize how the Aquivion case eventually lost CO_2_R selectivity, we performed ECSA, FIB, and spatial ICP-OES analysis. Between a pristine sample and after 80 min of electrolysis, the Aquivion sample showed an 11% decrease in capacitance as compared to the 25% drop of the bare copper over the same duration of electrolysis, indicating greater catalytic surface area at the same operating time (Figure S23). The ICP-OES data also showed no spatial copper difference after 80 or 375 min of operation (Figure 3e and Tab. S4), indicating, unlike the bare copper case, net in-plane migration of copper is inhibited. Lastly, an FIB analysis can be seen in Figure S24, showing the presence of Aquivion on top of the copper after 375 min of electrolysis. Again, we are unable to draw conclusions about how the average copper layer thickness changes in time.

An extension of the ionomer case is also the addition of a conductive overlayer, which provides a more even potential and current distribution across the catalyst layer. In turn, the in-plane directional migration of copper can be minimized. Here we then utilize the same base case of copper sputtered onto a PTFE GDL but then with carbon NPs sprayed on top with Aquivion as a binder. As a result, the operational lifetime of the 3 tested carbon NPs cases then shows a marked improvement in stability versus the bare copper electrode, even though the amount, distribution, and morphology of the underlying copper catalyst remain the same. Here operation reaches 6 h before the peak observed ethylene FE drops by >5%. Even 10–20% ethylene FE remains at 12 h, in stark contrast to the bare copper case (Figure S25). Here the ICP-OES of two individual carbon NP-coated copper GDEs showed no net in-plane copper migration after 80 or 465 min of operation (Table S5 and Figure 3f), indicating the failure mechanism is different from that of bare copper. In FIB characterization, some separation of the copper and carbon layers is evident, with the void containing electron beam sensitive materials such as ionomers (Figure S26). It is unclear if the gap between copper and carbon is a result of detachment during or after operation or copper restructuring that resulted in segregation.

Regarding the reason for the extended ethylene production, we again expect an increased local pH to be important. Both the ionomer from the deposition process and the addition of a porous overlayer could restrict the transport of hydroxide/carbonate from the copper surface. The porous overlayer also physically restricts the movement of any dissolved Cu-complexes. Finally, the carbon layer improves catalyst layer conductivity, which would reduce potential gradients, the directional migration force. Combined these effects explain why no net in-plane material movement is observed.

A question that remains then is how the ionomer and carbon NP cases are failing, even though lifetimes are improved. Here we can probe the overall data set to form a hypothesis. First, all scenarios showed similar product spectra in the early electrolysis period (Figure S25, S27–S28), indicating each catalyst layer had similar electrochemical behavior but with different longevities. In the ionomer and carbon NP cases, we also observed a measurable, but broader, temporary increase in methane FE (1–2%) corresponding to declines in ethylene. Notably repeated experiments also show low variability in product distribution over time (Figure S29), indicating a consistent failure mechanism. Finally, we did not observe any salt formation or flooding on the back side of the PTFE GDE, but we cannot explicitly rule out salt formation within the copper layer. We consider this unlikely to be the main failure mechanism; however, as the most physically confined systems show the greatest longevity. We also rule out surface facet changes as a destabilization mechanism as all copper layers are sputtered similarly and a big difference in achievable stability is observed between a 1.0 mg·cm^–2^ and 0.1 mg·cm^–2^ carbon overlayer on top of a sputtered Cu electrode (Figure S30). Observations of bubbles leaving the catholyte tube after loss of ethylene selectivity (Figure S31) also indicate that H_2_ evolution is occurring on the outer copper or carbon surfaces, rather than the buried copper ones. Such observation of H_2_ bubbles in the catholyte are common in the cases of CO_2_ starvation. Figure S32 compares the FE of hydrogen with and without taking the hydrogen leaving through the electrolyte into consideration. These combined observations lead us to hypothesize that the ionomer and carbon NP cases are then still failing through limitations of CO_2_ access, but this time the porous copper layer is becoming more evenly compact across the entire electrode instead of only at the perimeter.

Figure S33a shows linear sweep voltammetry curves from the OCP to −2.5 V. vs RHE of the bare Cu electrode, Aquivion-coated Cu, and carbon NP-coated Cu electrode before and after reaching failure under either N_2_ or air flow (Supporting Information). Each curve in Figure S33a represents the fifth or higher linear sweep voltammetry cycle number (Figure S34). Here the oxygen reduction reaction (ORR) current density profile can be observed, giving an indication of the gas accessibility from the gas channel to the electrolyte-immersed catalyst layer. As shown in Figure S33b and Table S6, the samples that underwent CO2ER show a large decrease in limiting current density, with the Aquivion and carbon overlayer samples showing a ∼3-fold reduction in ORR limiting current density compared to the pristine sample. These results indicate a more compact copper structure after operation in comparison to that of the bare Cu electrode.

A natural extension of the stabilizing features from Figure 2 is inherent in membrane electrode assemblies (MEA) shown in Figure 4a. An MEA cell excludes the use of a catholyte, resulting in a physically confined catalyst layer, higher local pH gradients, and much improved current distribution through a conductive flow field and carbon GDL. Because of the change in GDE and current collectors, copper sites are much closer to the current collector than in the PTFE case (Table S7–S8). We then took our 500 nm sputtered copper base case, applied it to a carbon GDL, and operated it at the same current density in an MEA cell (Figure S35). Here an anolyte of 0.1 M CsHCO_3_ is used to prevent salt precipitation; subsequently, no salt was observed after operation (Figure S36–S37).^20^ Within the MEA cell, the bare copper case, with no ionomer or carbon NPs, is then able to produce ethylene FEs > 35% for 12 h, as compared to the <1 h in a flow cell with a PTFE GDL (Figure 4b). Here the test was stopped at 12 h due to increasing cell potentials (Figure S38) and the dissolution of the Piperion membrane (Figure S39), which we attribute to byproduct ethanol.^12^ With the aim of mitigating membrane dissolution, we added a 0.1 mg·cm^–2^ carbon NPs layer on top of copper to lower ethanol crossover, but membrane dissolution occurred again after 15 h (Figure S40).

**Figure 4:**
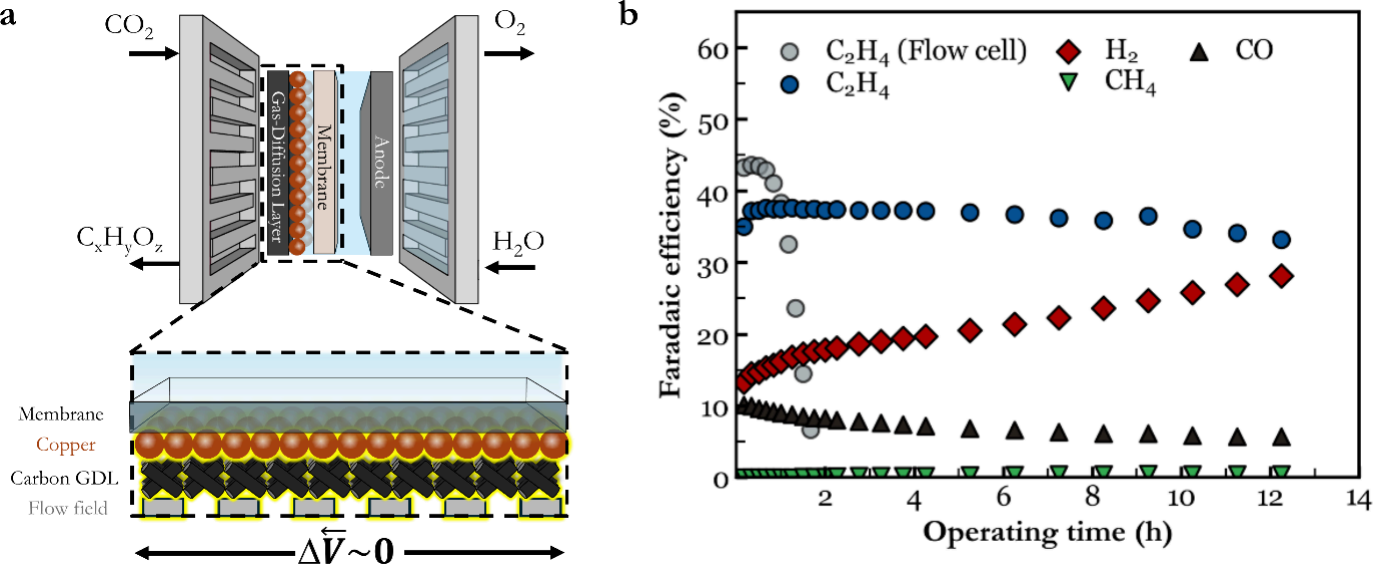
(a) Cell architecture of an MEA cell and a 500 nm sputtered copper catalyst on a carbon GDL. The metallic flow field acts as a current collector, minimizing potential gradients across the copper layer. (b) FE profiles of gas products comparing the flow-cell PTFE architecture with the MEA architecture at −200 mA·cm^–2^ by using a copper sputtered carbon GDL.

Combined, these results show how instability results from the dissolution-redeposition cycles that are inherent to CO_2_R and that instability can be mitigated through increasing conductivity and confinement. We then provide a missing link between copper catalyst restructuring and selectivity changes over time, illustrating that microscale restructuring is a dominant mechanism for the failure of the CO_2_R mechanism. Specifically, we show that the failure mechanism for pure copper systems in a flow-cell system occurs via in-plane copper migration. Improvements in the catalyst layer architecture then shift the in-plane failure mechanism to through-plane copper layer compaction, leading to an elongated operation characterized by steady CO_2_ starvation. These findings provide a mechanistic reason why MEA cells with low potential gradients demonstrate the longest ethylene stabilities in literature, despite elevated current densities and challenges with flooding and salt precipitation.^19–22,67^ Finally, extending our observations toward >10,000 h of operation indicates that MEA configurations are still likely to face an uphill battle against catalyst layer compaction. Micropotential gradients will always exist across the catalyst layer, providing a slow driving force for copper migration which may only be realized over extended operation. A built-in or operational mechanism for periodic copper redistribution may then be necessary in the future.^72^

## Supplementary Material



## Data Availability

All data is made available in the manuscript and the Supporting Information. Raw data made available through deposition on the 4TU.Centre.

## Notes

Materials and methods; results of bare copper electrode operation; results of copper electrode with overlayers; results of MEA test (PDF)
