# Supplementary material for: Role of the Copper Microstructure on Ethylene Stability during CO2 Electrolysis
Source: ACS Energy Lett. 2026 Mar 11;11(4):3633–41. doi: 10.1021/acsenergylett.6c00513 (PMC13077678; doi:10.1021/acsenergylett.6c00513)
Supplement: Supplementary file 1 [file nz6c00513_si_001.pdf]

Supplementary Materials for

The role of the copper microstructure on ethylene stability during CO_2 electrolysis

Jesse Kok[1,2†], Nikita Kolobov[1,2†], Mohammed Sharah[1,2], Amir Foroozan[3], Shayan Angizi[3], Konstantinos Dimitriou[1,2], Drew Higgins[3], Thomas Burdyny[1,2*]

*Corresponding author email: T.E.Burdyny@tudelft.nl

[1] Department of Chemical Engineering, Delft University of Technology, van der Maasweg 9, 2629 HZ, Delft, The Netherlands

[2] e-Refinery Institute, Delft University of Technology, Leeghwaterstraat 39, 2628 CB Delft, The Netherlands

[3] Department of Chemical Engineering, McMaster University, Hamilton, Ontario L8S 4L7, Canada.

† Jesse Kok and Nikita Kolobov contributed equally as first-authors.

Table of contents

Materials and methods 4

Cathode preparation 4

CO_2 electrolysis in flow cell 5

CO_2 electrolysis in MEA cell 6

Product analysis 7

Characterisation 8

Capacitance Measurements 10

Calculation of limiting O_2 reduction reaction (ORR) current density 10

Results bare copper electrode operation 12

Figure S1. (a) SEM image of a PTFE substrate. (b) SEM image of a 500 nm copper sputtered PTFE. ..12

Figure S2. Experimental setup used to perform experiments with the PEEK flow cell. 12

Figure S3. Components inside the PEEK flow cell. 13

Figure S4. Uncompensated cathode potential (V vs RHE) of the copper GDE. 14

Figure S5. The Faradaic efficiency profiles of gas products during four independent CO2 electroreduction experiments 15

Figure S6. Full product distributions of a bare copper GDE 16

Figure S7. Average current as a function of scan rate before operation and after catalyst failure 16

Table S1. Capacitance values before operation and after failure of bare copper GDE (see Fig. S10). 17

Figure S8. Back of copper GDE after operation with an absence of visible salt particles and electrolyte. 17

Figure S9. The Faradaic efficiency profiles of the gas products up to the point at which the copper GDE was taken out of the flow cell for SEM analysis. 18

Figure S10. SEM imaging taken of an unused copper sample. 19

Figure S11. SEM imaging taken of the copper sample after 80 minutes. 19

Table S2. ICP-OES data of pristine bare copper sample and failed (80 minutes of operation) bare copper sample. 20

Figure S12. FIB analysis revealing the cross-sectional morphology of the center and perimeter of the copper GDE 20

Figure S13. Changing current collector shape. 21

Figure S14. Uncompensated cathode potential (V vs RHE) of the copper GDE with a y-shaped current collector 22

Figure S15. Postmortem SEM imaging at three different locations for the y-shaped current collector 22
Figure S16. Faradaic efficiency profiles of gas products as a function of operating time........ 23
Figure S17. Buffer capacity comparison between the Faradaic efficiencies of ethylene........ 23
Results copper electrode with overlayers........ 24
Figure S18. Uncompensated cathode potential as a function of operating time 24
Figure S19. Faradaic efficiency of ethylene as a function of operating time 25
Figure S20. Faradaic efficiency profiles of gas products as a function of operating time........ 26
Figure S21. Faradaic efficiency of ethylene as a function of operating time 26
Figure S22. Open-circuit profile for different electrodes as a result of the local hydroxide-concentration as a function of time. 27
Figure S23. Average current as a function of scan rate 27
Table S3. Capacitance values........ 28
Table S4. ICP-OES data of Aquivion® coated copper sample 28
Figure S24. FIB analysis revealing the cross-sectional morphology........ 29
Table S5. ICP-OES data of carbon NPs coated copper sample 29
Figure S25. The Faradaic efficiency profiles of gas products during five independent CO_2 electroreduction experiments........ 30
Figure S26. FIB analysis revealing the cross-sectional morphology........ 32
Figure S27. The Faradaic efficiency profiles of gas products during six independent CO_2 electroreduction experiments........ 32
Figure S28. Full product distribution of different GDEs. 34
Figure S29. Average Faradaic efficiency profiles of gas products as a function of time........ 34
Figure S30. Faradaic efficiency profile of ethylene as a function of operating time for different loadings of carbon NPs on top of a sputtered copper catalyst. 35
Figure S31. Hydrogen bubble formation in catholyte tubes 35
Figure S32. Faradaic efficiency profiles of hydrogen with and without taking the hydrogen leaving the flow cell through the electrolyte into consideration. 36
Figure S33. Results of oxygen reduction linear sweep voltammetry for various Cu samples using N_2 or air flow in the gas channel of the electrochemical cell........ 38
Figure S34. Multiple cycles were needed before the curves within the oxygen reduction reaction potential range started to overlap. 38
Table S6. Approximate oxygen reduction reaction (ORR) limiting current densities observed for different gas flow and catalyst architectures via linear sweep voltammetry (LSV)........ 38
Results MEA test 39

Table S7. Flow cell works reporting their current collector dimensions and stability. 39
Table S8. MEA cells reporting their current collector dimensions and stability. 39
Figure S35. MEA cell experimental setup 39
Figure S36. Image showing absence of salt particles 40
Figure S37. Image showing the cathode serpentine flow field pattern... 41
Figure S38. Potential as a function of operating time 42
Figure S39. PiperION® membrane post stability test showing cavity. 43
Figure S40. FE profiles of gas product produced during stability test 44

Materials and methods

Cathode preparation

We fabricated copper (Cu) gas diffusion electrodes (GDE) through magnetron sputtering a polytetrafluorethylene (PTFE) substrate (0.45 µm pore size with 25 µm layer thickness, Sterlitech) under rotation at an operating pressure of 3µbar. The sputtering time and sputter gun power were regulated to achieve the desired 500 nm flat thickness. In case of coated Cu GDEs, the coated layers of ionomers and carbon nanoparticles (NPs) were deposited using spray coating (Harder & Steenbeck, Evolution). The ink used for spraying carbon NPs consisted of 12 mg of Carbon NPs (Vulcan XC72R, Cabot, 30 to 60 nm), 4 mL of methanol (Sigma Merck) and 20 microliter of a Aquivion® CEI (25 wt% in water, 790 g/mol SO_3H Sigma Merck). 4 mL of the ink was sprayed on top of a 1.8 cm x 1.8 cm Cu GDE to reach a 1 mg/cm^2 carbon NPs loading. During this procedure, the Cu GDE was fixed to a 65 °C heating plate. For the deposition of an Aquivion® ionomer, 0.25 mL of Aquivion® 25 wt% was dissolved in 6 mL of methanol. Subsequently, 0.5 mL of this solution was sprayed on top of the Cu GDE placed on a 35 °C heating plate. The same

heating conditions were applied for a Sustainion® (XA-9 5% in ethanol) coating. To create the coating mixture, 0.2 mL from the Sustainion® 5% solution was dissolved in 5 mL of methanol. Subsequently, 0.25 mL of this solution was used for spraying. Prior to spraying, each solution was sonicated for at least 30 minutes. Following the spray coating, the samples were dried in a vacuum oven overnight. The 500 nm Cu GDE tested in the membrane electrode assembly (MEA) cell was prepared by magnetic sputtering a Sigracet 39 BB gas diffusion layer (GDL).

CO_2 electrolysis in flow cell

All of the flow cell experiments were done in a 2.25 cm^2 Polyetheretherketon (PEEK) flow cell. An image of the disassembled flow cell is shown in the Fig. S3. The cell consisted of an anolyte chamber, catholyte chamber and gas chamber. A 1 M $KHCO_3$ (Sigma Merck) solution was circulated at 20 mL/min to both the anolyte chamber and catholyte chamber by two peristaltic pumps (MasterFlex). A 1 M $KHCO_3$ electrolyte was used in every experiment unless stated otherwise. Two sets of dampers were installed to reduce cyclic pressure spikes caused by the peristaltic motion of the pumps. The anolyte and catholyte were separated by a cation exchange membrane (CEM) Nafion 115 (Ion Power). A PTFE Cu GDE, a IrO_x/RuO_x mesh (De Nora) and a reversible hydrogen electrode (Gaskatel), were utilised as cathode, anode and reference electrode, respectively. A Cricut 3 cut copper tape was applied as a current collector. The insides of the flow cell were sealed with gaskets and the complete setup was tightened to 1.5 Nm torque. Carbon dioxide (CO_2) was supplied at 40 sccm to the backside of the Cu GDE through the gas chamber channels by a mass flow controller (Bronkhorst). Potentials were supplied using a Parstat4000 potentiostat (Princeton Applied Research). During operation, gas and liquid pressures were

controlled and monitored using a BPR system (Bronkhorst). The gas flow rate was measured by a mass flow meter, and its content was analysed using a gas chromatography (GC, Global Analyser Solutions® CompactGC 4.0). A liquid trap was positioned between the flow cell and the GC to prevent any liquid droplets from entering the GC. Visual descriptions of the setup are shown in Fig. S2 and Fig. S3

CO_2 electrolysis in MEA cell

A CO_2 electrolysis stability test was performed in a 5.06 cm^2 membrane electrode assembly (MEA) cell. The MEA comprised a titanium block anode with a pin type flow channel and a stainless steel cathode compartment with a serpentine flow channel. The two compartments were separated by a 40 µm piperion (Vitrogen) anion exchange membrane (AEM). The membrane was activated by soaking it for 48 hours in 1 M KOH. After 24 hours, the solution was replaced by a fresh 1 M KOH. A 0.1 M $CsHCO_3$ (Sigma Merck) anolyte was circulated at 20 mL/min by a peristaltic pump (MasterFlex). A IrO_x on Ti felt (0.6 $mg \cdot cm^{-2}$, 200 µm, HPNow) and a Sigracet 39 BB carbon GDL sputtered Cu were employed as anode and cathode, respectively. In a second MEA stability test, the Cu GDE was coated with 0.25 $mg \cdot cm^{-2}$ off carbon NPs. Both the anode and the cathode were supported by a PTFE gasket. The titanium and stainless steel blocks were pressed together to a 2.5 Nm torque. CO_2 was supplied at 50 sccm to a humidifier and controlled by the mass flow controller (Bronkhorst). Potentials were supplied to the MEA cell using a Parstat4000 potentiostat (Princeton Applied Research). The outlet gas flow rate was measured with a mass flow meter and its content was analysed using a gas chromatography (GC, Global Analyser Solutions® CompactGC 4.0). A liquid trap was employed to prohibit liquid droplets from entering the GC.

For a more detailed description of the setup used for CO_2 electrolysis in both flow cell and MEA-cells, we would like to refer the reader to a published guide on the assembly and operations of these cell architectures[1]. A visual description of the setup is shown in Fig. S30.

Product analysis

Both gas and liquid products were analysed to compute the selectivity. Gas products were quantified using an online GC. Here, every 10, 15, 30, 60 or 120 minutes of operation, a 50 microliter injection was taken from the outlet gas channel to analyse its contents. A calibration file was made to calculate the concentration of ethylene, methane, hydrogen, and carbon monoxide in parts per million (ppm).

The concentration (C_x), along with the measured gas flow rate (V_{total}), is then used to compute the Faradaic efficiency (FE_x) of component 'x' as shown in the following equation:

$$FE_x = \frac{C_x \cdot 10^{-6} \cdot V_{total} \cdot 10^{-6} \cdot \frac{P}{R \cdot T} \cdot n \cdot F}{i \cdot 60} \qquad (1)$$

Here, C_x was expressed in units of PPM (parts per million), the measured flow rate (V_{total}) in $mL \cdot min^{-1}$, the pressure (P) and temperature (T) were set to 101325 Pa and 273 K, respectively, n is the number of electrons consumed in the reduction reaction for the formation of product x, F is Faraday's constant (96485.3 C/mol) and i is the applied current in A. The measured flow rate was corrected as the mass flow meter was calibrated for CO_2 only. This was done using a gas conversion factor. See equations below.

$$V_{total,corrected} = \frac{V_{total,measured}}{0.74} \cdot C_{mix} \qquad (2)$$

$$C_{mix}^{-1} = \sum_{i=1}^{6} \frac{y_x}{\text{Gas conversion factor}} \qquad (3)$$

The gas correction factor is 0.74 for CO_2, 0.79 for water vapour, 0.6 for ethylene 0.7 for methane, 1.0 for carbon monoxide, and 1.01 for hydrogen.

Liquid products (ethanol, propanol, formate and acetate) were quantified using ^{1}H-NMR (Agilent 400 MHz). A Ø 5 mm nmr tube was filled with a 550 μL diluted sample of the electrolyte and 50 μL of a 50 mM maleic acid (Sigma Merck) in D_2O (Sigma Merck) solution. The equation below was then used to compute the faradaic efficiencies of the liquid products.

$$\mathrm{FE_x} = 100 \cdot \frac{\left(\frac{\mathrm{I_x}}{\mathrm{I_{std}}} \cdot \frac{\mathrm{N_{std}}}{\mathrm{N_x}} \mathrm{C_{std}} \cdot \frac{\mathrm{V_{tube}} \cdot \mathrm{V_{electrolyte}}}{\mathrm{V_{sample}}} \cdot \mathrm{n} \cdot \mathrm{F}\right)}{(\mathrm{i} \cdot \mathrm{t})} \quad (4)$$

Here, I_x and I_{std} are the integrals of the liquid compound x and the internal standard (maleic acid) as determined by the Mestrenova software, respectively. C_{std} is the concentration of the internal standard in the NMR tube in mol·L^{-1}. V_{tube}, $V_{electrolyte}$, and V_{sample} are the NMR tube volume (L), electrolyte volume (L) and volume of the electrolyte sample in the NMR tube (L), respectively. Finally, t is the experimental time.

Characterisation

Cu GDEs, bare and coated, were taken out at different operational timescales and studied using *ex situ* scanning electron microscopy (SEM), focused ion beam (FIB) and localized Inductively-Coupled Plasma Optical Emission Spectroscopy (ICP-OES).

Ex situ SEM analysis was performed in a JEOL JMS-IT700 instrument. After removal of the Cu GDE from the flow cell, the sample was cleaned with Milli-Q water and subsequently dried in a vacuum oven. Several images were taken at different magnifications of both the perimeter and the center of the Cu GDE.

To prepare for a cross-sectional view, the samples were mounted on Al SEM stubs by applying silver paste (TED PELLA, INC) to the edges and creating a conductive path from the Cu to the stub. Then the samples were placed in an oven at 60 °C for 15 min to dry the paste before entering the vacuum chamber of the microscope (Zeiss Crossbeam 350). To protect the top layer from ion beam damage and obtain an intact cross section, first, a thin layer of tungsten (~ 100 nm) was deposited by flowing Tungsten hexacarbonyl $W(CO)_6$, gas over the area of interest and exposing it to the electron beam at 3 kV. A low voltage was selected to minimize the penetration of W deep in the pores of the sample. Then a thicker layer of W (~ 2 microns) was deposited using gallium (Ga) ion beam and flowing the same gas over the area of interest. The FIB using Ga ions was used to produce cross sections of the electrodes. A final FIB step was applied at low energy to obtain a smooth and intact view of the cross-section which enabled to observe the distinction between the various grains of the Cu phase. Secondary electrons were used for image acquisition resulting in heavier elements to appear brighter than lighter elements.

ICP analysis performed on the catalyst layer material taken from the perimeter and center of the various GDEs samples gave insights into the occurring microstructural change. Circles with a 3 mm diameter were punched out of the different GDEs prior or after undergoing CO_2 electrolysis. Two of these circular samples were taken from both the perimeter and center in order for the measured ppm range to fall within the maximum resolution.

Subsequently, the samples were brought into a Teflon-lined vials containing 10 mL of aqua regia (1 vol% HNO_3 : 2 vol% HCl) and heat up to 75 °C and maintained at this temperature for 48 hours while being continuously stirred. All solutions were filtered using a 0.22 µm PTFE syringe filter to remove any solid residues and prevent particle contamination of the spectrometer chamber.

The ICP data is based on an average out of 3 distinct copper signals (213.598 nm, 324.754 nm, 327.395 nm).

Capacitance Measurements

The working electrode capacitance was calculated by taking several cyclic voltammograms over a non-faradaic region at different sweep rates. The chosen non-faradaic window covered 0.3 V vs RHE to -0.05 V vs RHE. The chosen sweep rates consisted of 10, 20, 50, 65, 80, and 100 mV/s. The measured current was averaged over the entire potential window for both the cathodic sweep and anodic sweep. Accordingly, the average current over both sweep directions was computed and plotted as a function of sweep rate. A trendline was fitted to subsequently extract its slope. The calculated slope was defined as the capacitance of the working electrode. Due to slight deviations in non-linearity, the calculated capacitance values should be considered as approximate estimates.

Calculation of limiting O_2 reduction reaction (ORR) current density

The limiting O_2 reduction reaction current density on Cu electrodes and Cu electrodes with an Aquivion® and carbon NPs overlayer before operation and after reaching failure were evaluated based on multiple cycles of linear sweep voltammetry from OCP to -2.5 V vs RHE. We observed that scans equilibrated after five scans (see Fig. S34). Hence, the fifth scan for each scenario is shown in Fig. S33).

Either N_2 or air (21% O_2) was supplied at 40 sccm into the flow cell gas channel during the linear sweep voltammetry scans. As the potential required for the oxygen reduction reaction on a copper catalyst in 1 M $KHCO_3$ catholyte is more anodic than HER, a partial current density plateau is observable between -0.6 vs RHE and -1.25 V vs RHE before current increases again due to HER. The plateau is caused by a limiting O_2 flux to the active sites available for O_2 reduction, implying

that the limiting current density is proportional to the gas accessibility of O_2. Note that the potentials are uncorrected for ohmic drops as gas evolution and linear sweep voltammetry can lead to large overcorrections. Thus, while ORR is occurring predominantly at potentials more anodic than 0 V vs RHE, the uncorrected potentials are much larger. The distance from the working and reference electrodes is 7.5 mm and the electrolyte pH ~ 7.

Results bare copper electrode operation

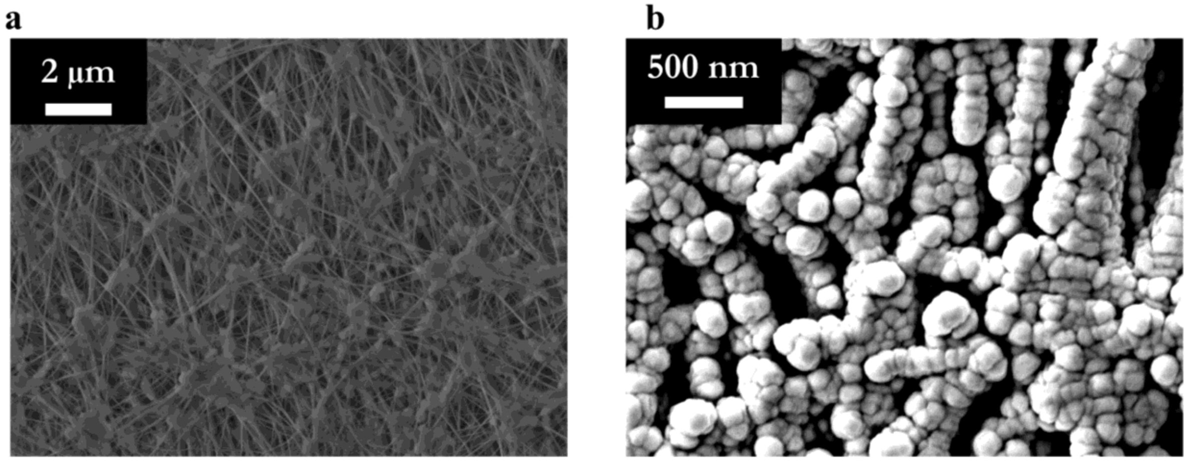

Figure S1. (a) SEM image of a PTFE substrate. (b) SEM image of a 500 nm copper sputtered PTFE.

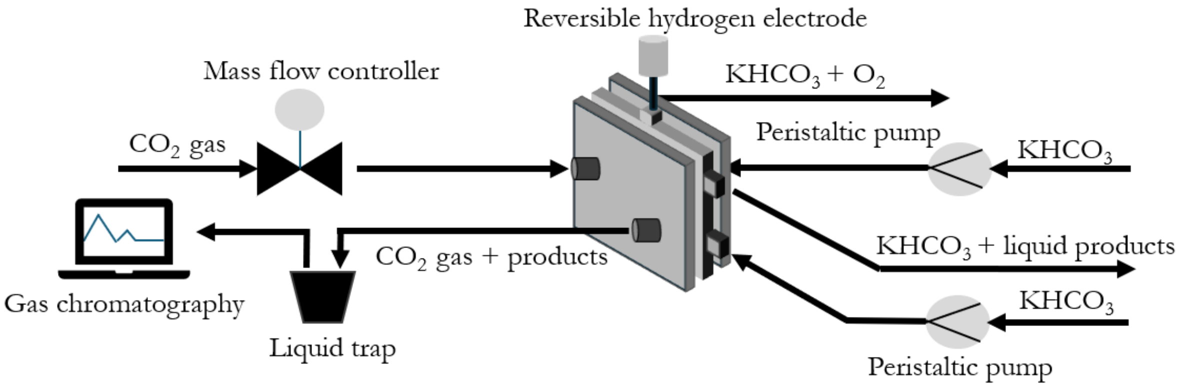

Figure S2. Experimental setup used to perform experiments with the PEEK flow cell.

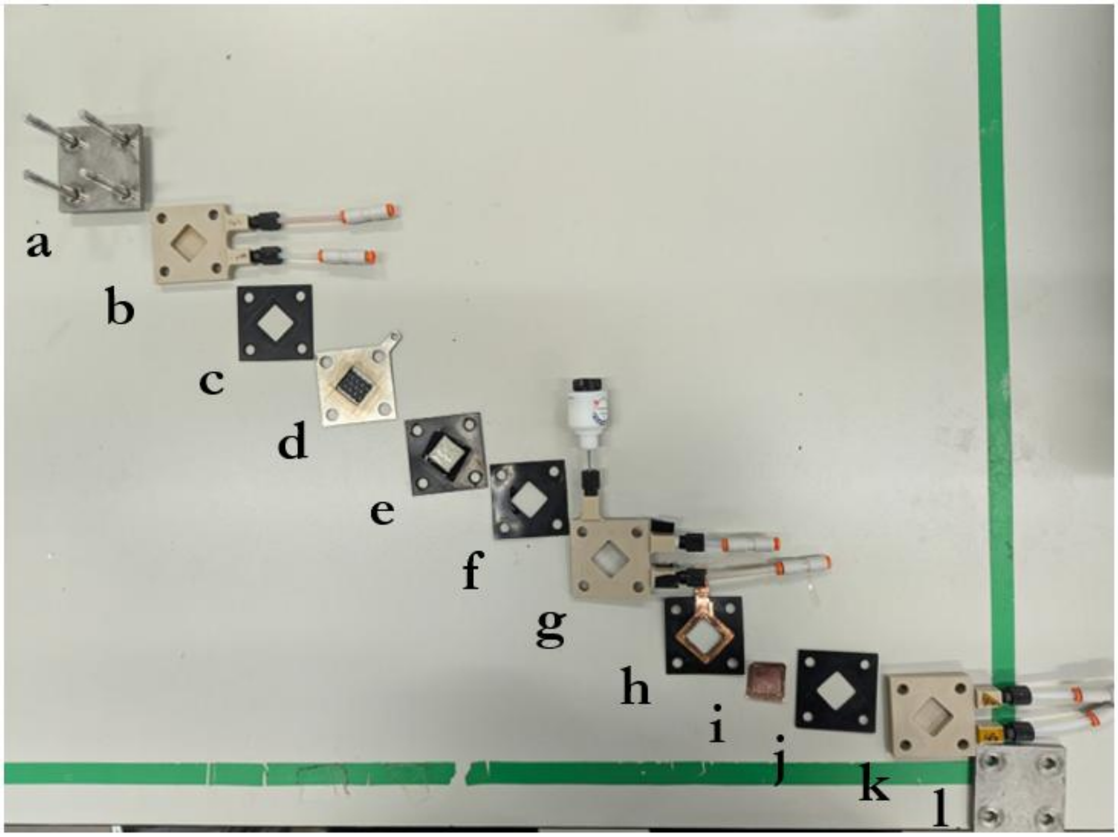

Figure S3. Components inside the PEEK flow cell. From top left to bottom right: (a) Metal plate with screws, (b) anolyte chamber, (c) gasket, (d) current collector with IrOx/RuOx mesh, (e) gasket with membrane, (f) gasket, (g) catholyte chamber with reversible hydrogen reference electrode, (h) gasket with current collector, (i) copper electrode, (j) gasket, (k) gas chamber, (l) metal plate.

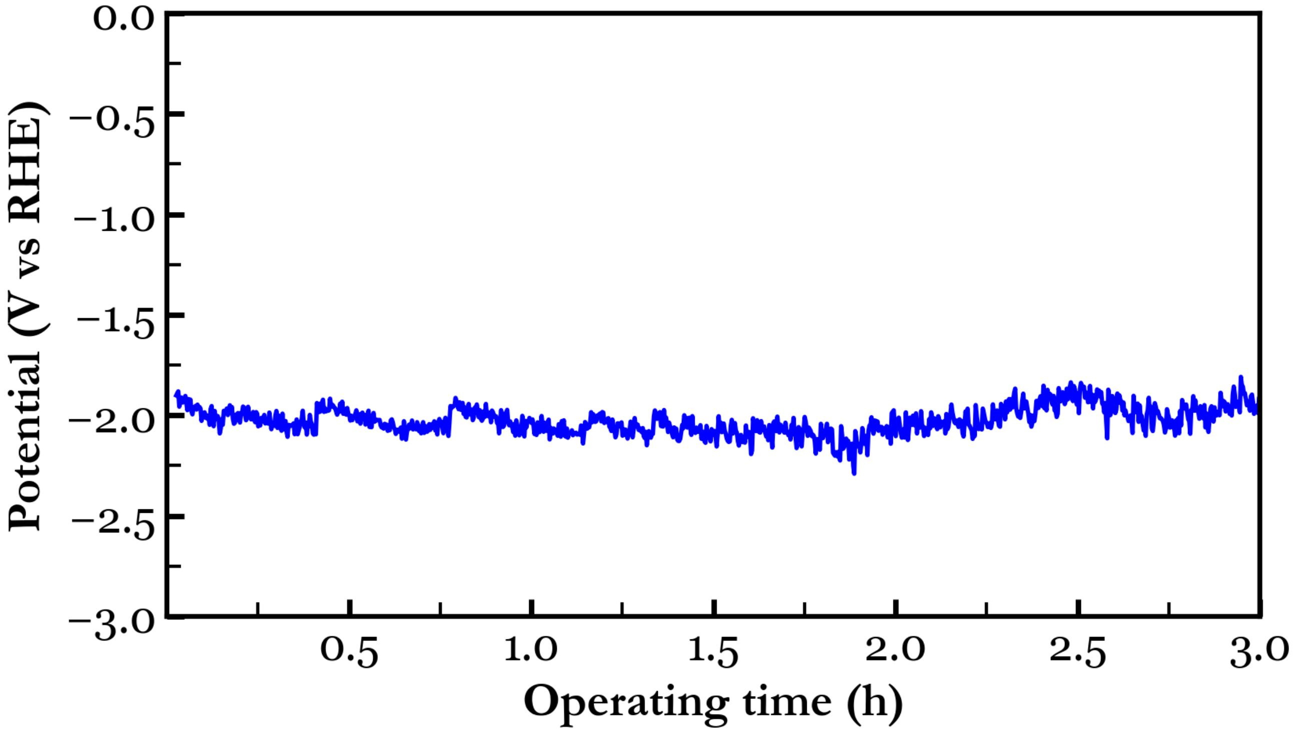

Figure S4. Uncompensated cathode potential (V vs RHE) of the copper GDE as a function of operating time at -200 mA·cm^{-2} in 1 M $KHCO_3$.

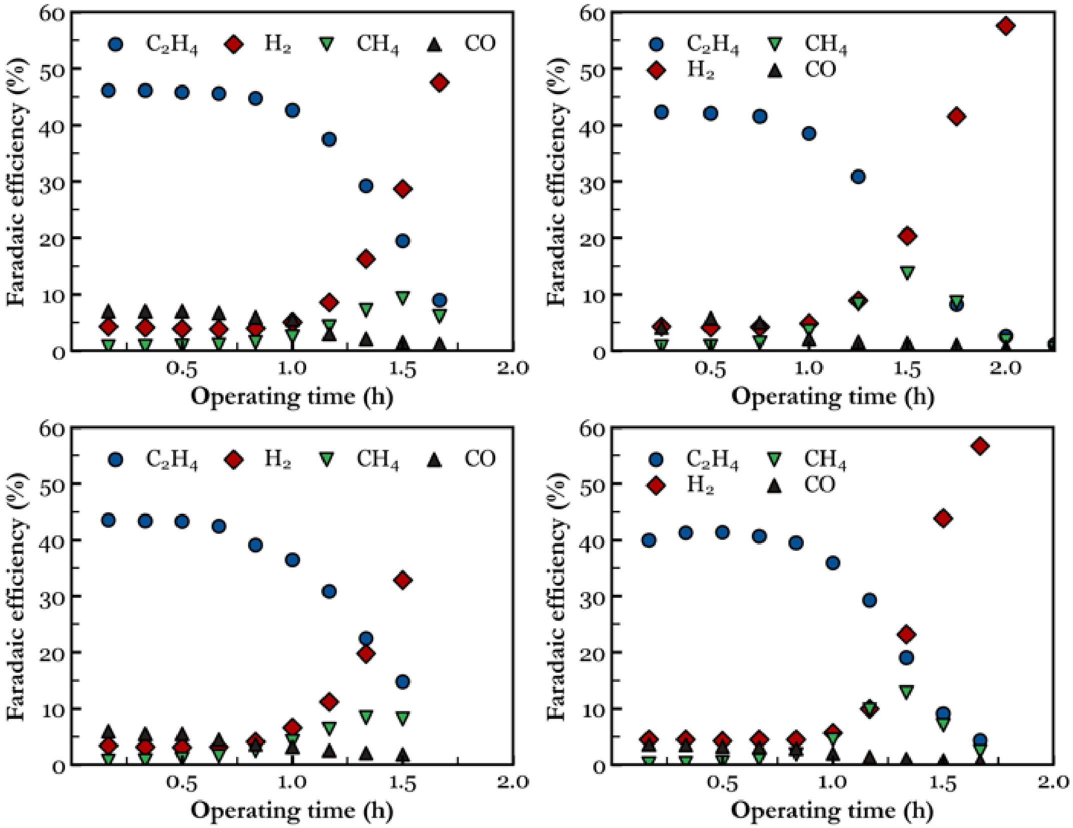

Figure S5. The Faradaic efficiency profiles of gas products during four independent CO_2 electroreduction experiment for a 500 nm copper layer sputtered onto PTFE in 1 M $KHCO_3$ at a current density of -200 $mA \cdot cm^{-2}$.

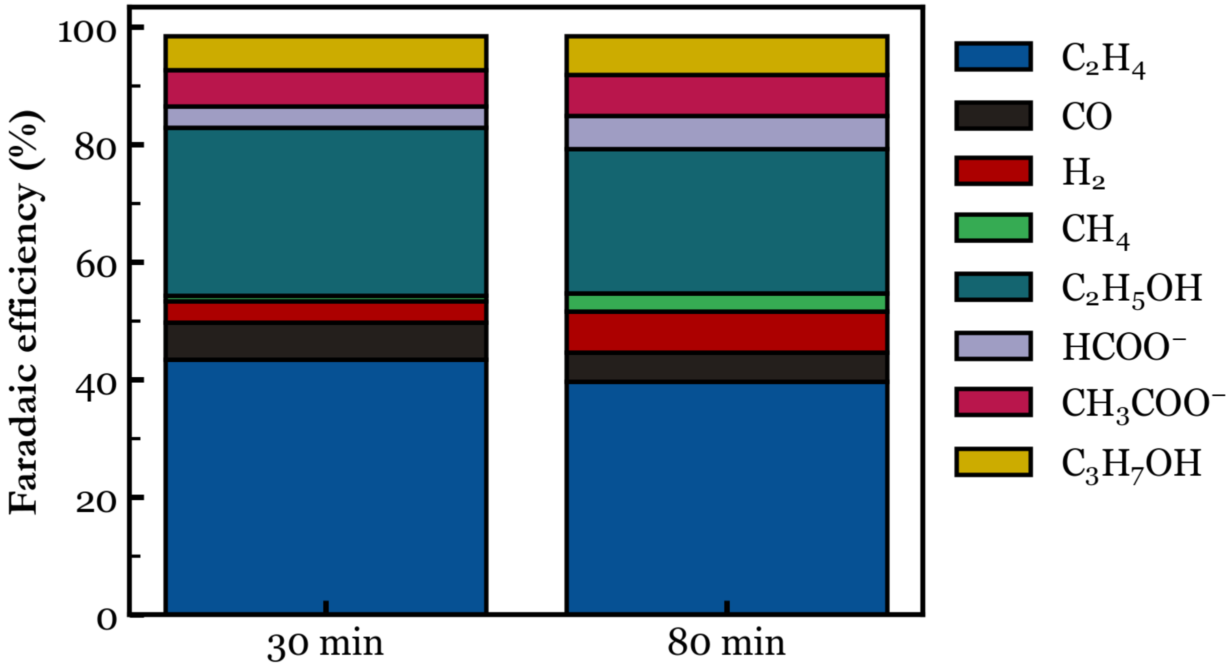

Figure S6. Full product distributions of a bare copper GDE after 30 minutes and 80 minutes of operation at -200 mA·cm^{-2} in a PEEK flow cell using 1 M $KHCO_3$ as catholyte.

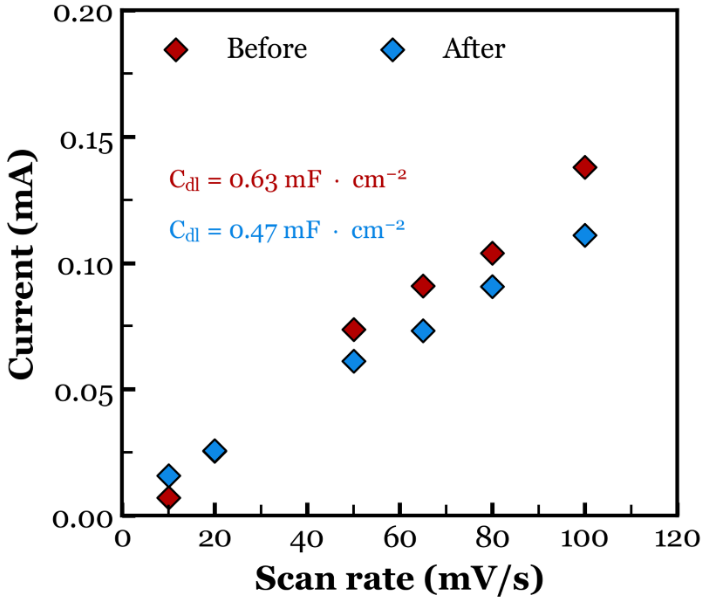

Figure S7. Average current as a function of scan rate before operation and after catalyst failure (80 minutes of operation) of the bare copper GDE. The slope of curve represents the electrode capacitance, and its value is given in Table S1.

Table S1. Capacitance values before operation and after failure of bare copper GDE (see Fig. S7).

Experimental time	Capacitance value ($mF \cdot cm^{-2}$)
Before operation	0.63
After 80 minutes of operation	0.47

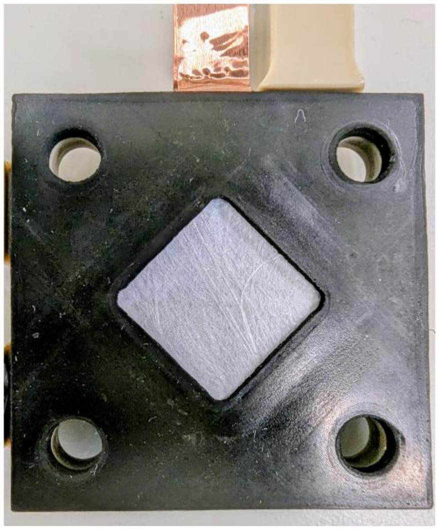

Figure S8. Back of copper GDE after operation with an absence of visible salt particles and electrolyte.

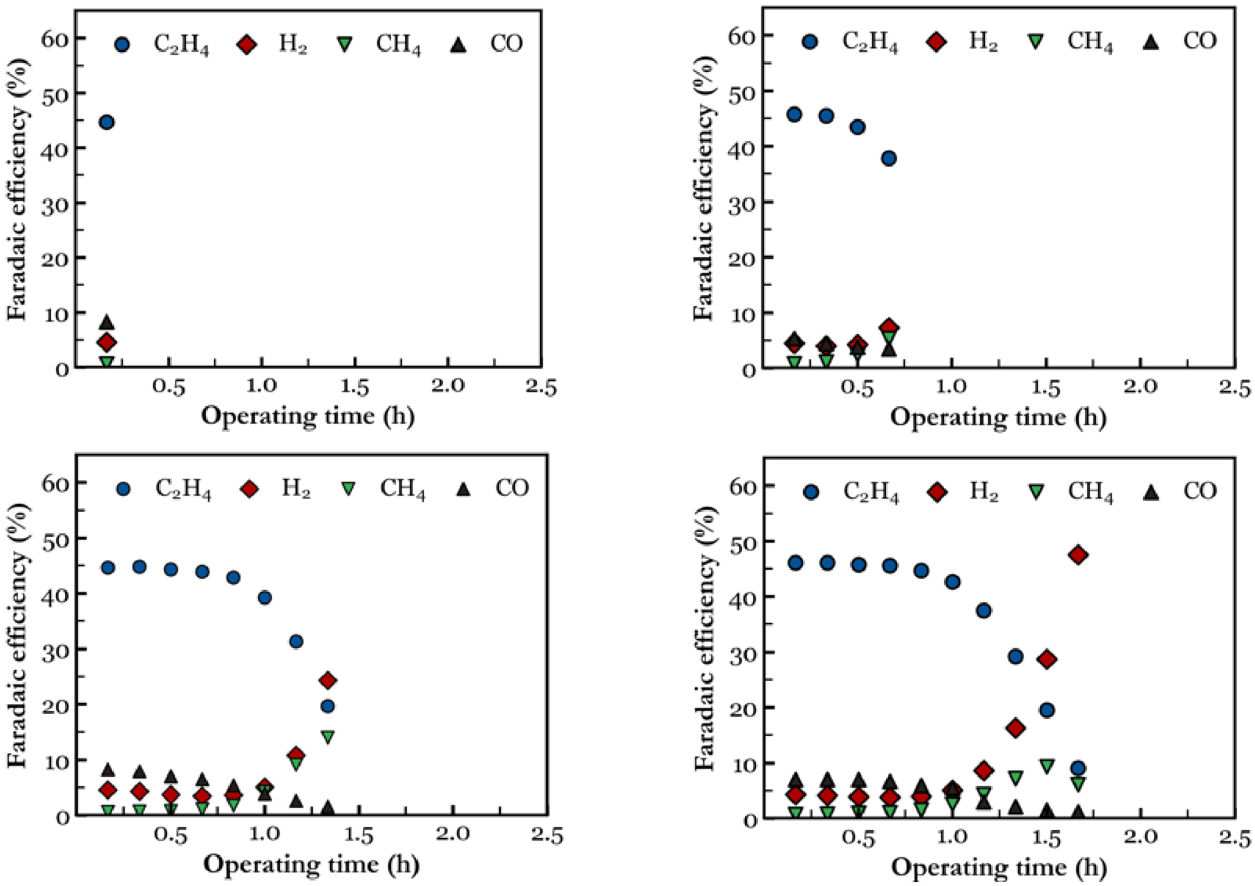

Figure S9. The Faradaic efficiency profiles of the gas products up to the point at which the copper GDE was taken out of the flow cell for SEM analysis. The samples correspond to the SEM images in Fig. 1b-c.

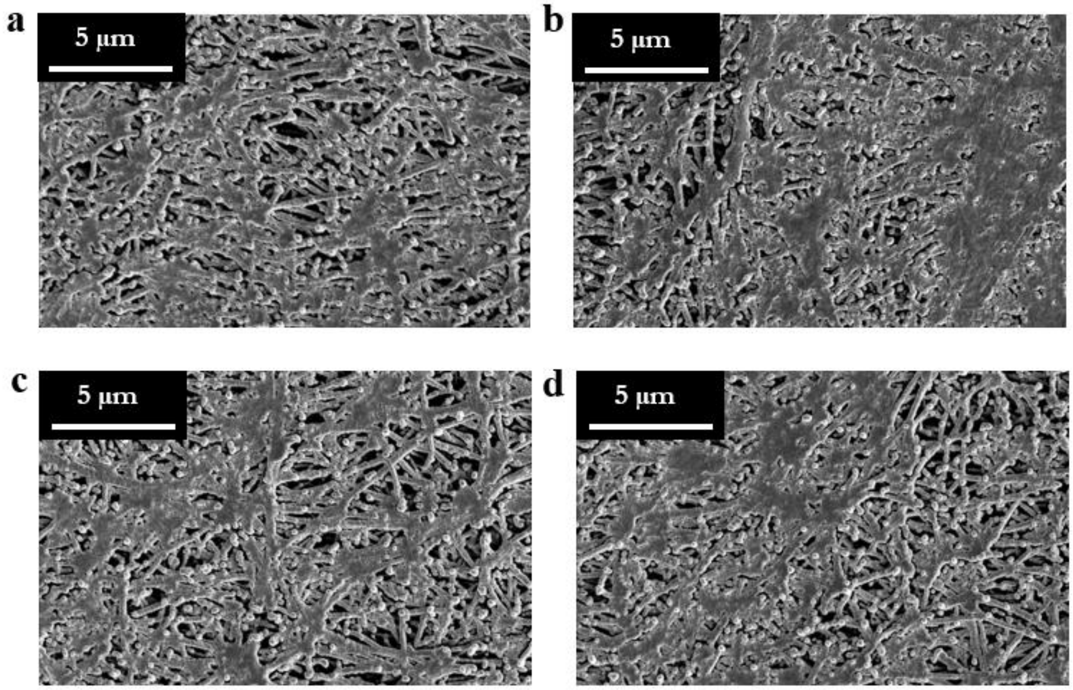

Figure S10. SEM imaging taken of an unused copper sample. (a-b) Center images. (c-d) Perimeter images

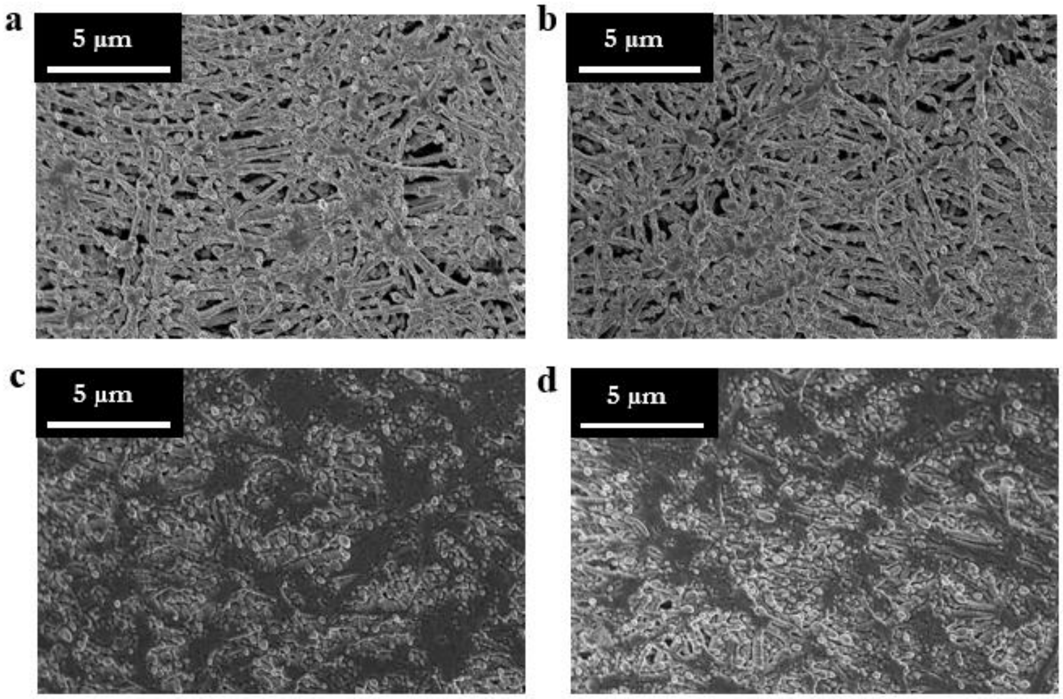

Figure S11. SEM imaging taken of the copper sample after 80 minutes. (a-b) Center images. (c-d) Perimeter images.

Table S2. ICP-OES data of pristine bare copper sample and failed (80 minutes of operation) bare copper sample. Bracketed numbers represent the wavelength used for the ppm determination.

Sample name	Sample Location	Copper (ppm) (213, 598 nm)	Copper (ppm) (324, 754 nm)	Copper (ppm) (327, 395 nm)	Average (ppm)
Pristine copper (before operation)	Perimeter	5.32	5.29	5.25	5.29
	Center	5.38	5.38	5.33	5.36
Copper after 80 minutes of operation	Perimeter	6.41	7.35	7.29	7.02
	Center	5.74	6.58	6.51	6.3

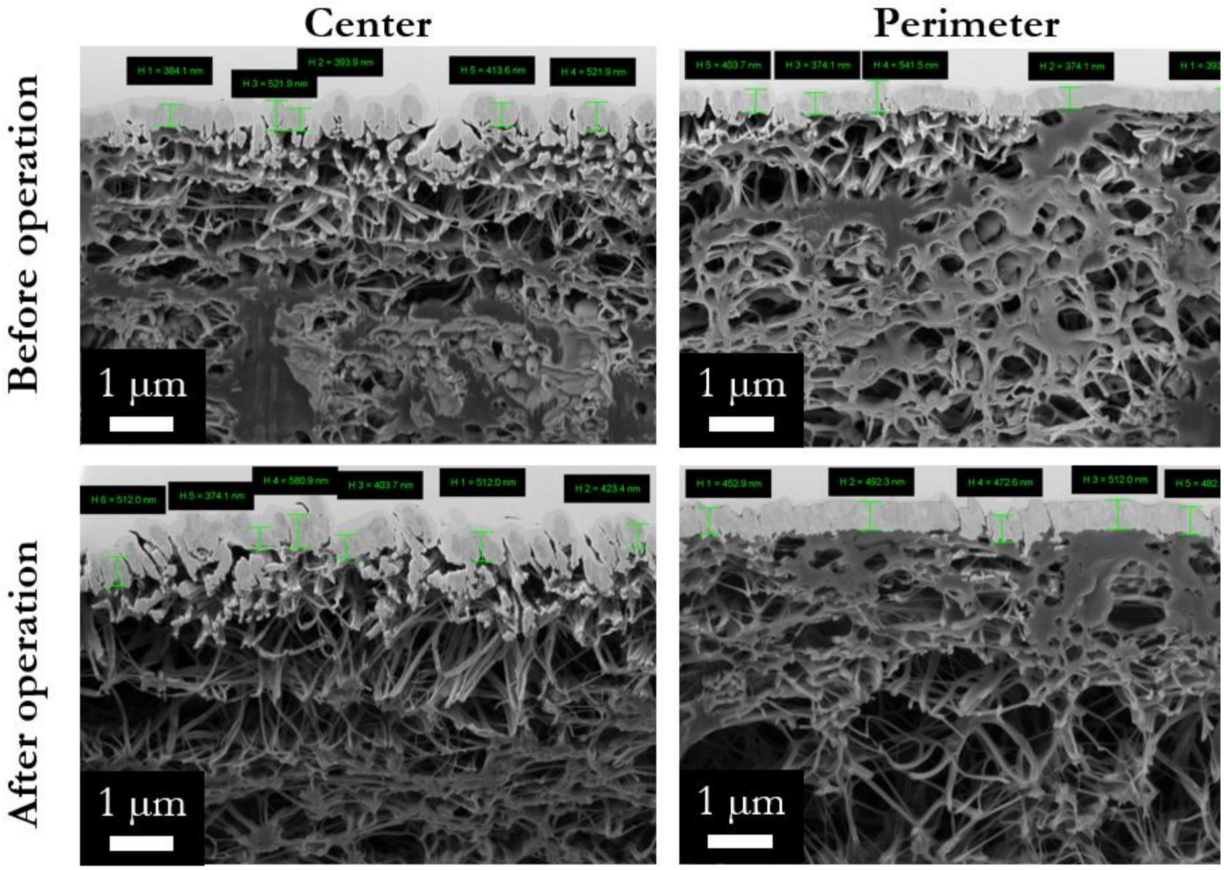

Figure S12. FIB analysis revealing the cross-sectional morphology of the center and perimeter of the copper GDE before and after 80 minutes of operation. Green bars indicate where thickness measurements were taken.

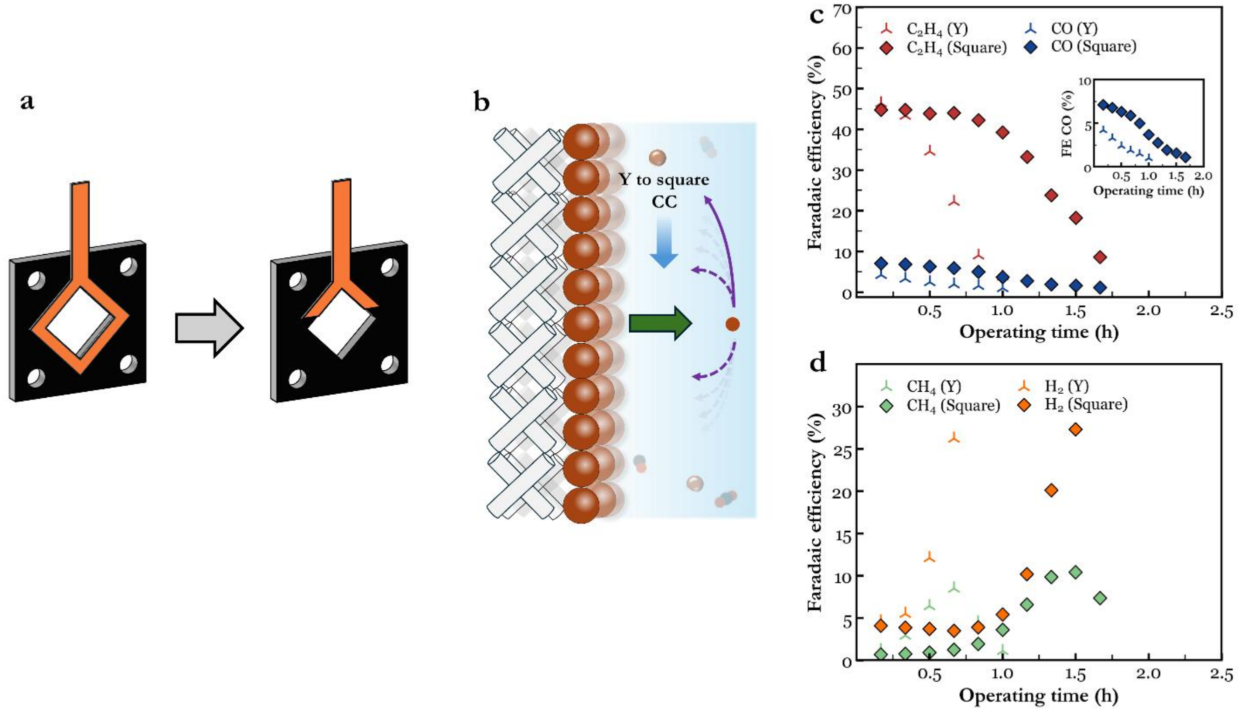

Figure S13. Changing current collector shape. (a) Change in current collector (CC) shape used to study potential gradient and catalyst lifetime. Here the squared-shape CC is replaced with a y-shaped CC. (b) Schematic highlighting the greater voltage gradient and subsequent faster copper migration for y-shaped CC (solid line) vs the square-shaped CC (dashed line). (c) Faradaic efficiency profiles of CO and C_2H_4 and (d) H_2 and CH_4 measured as a function of operating time for a copper GDE subjected to -200 $mA{\cdot}cm^{-2}$ using the square-shaped and y-shaped CC.

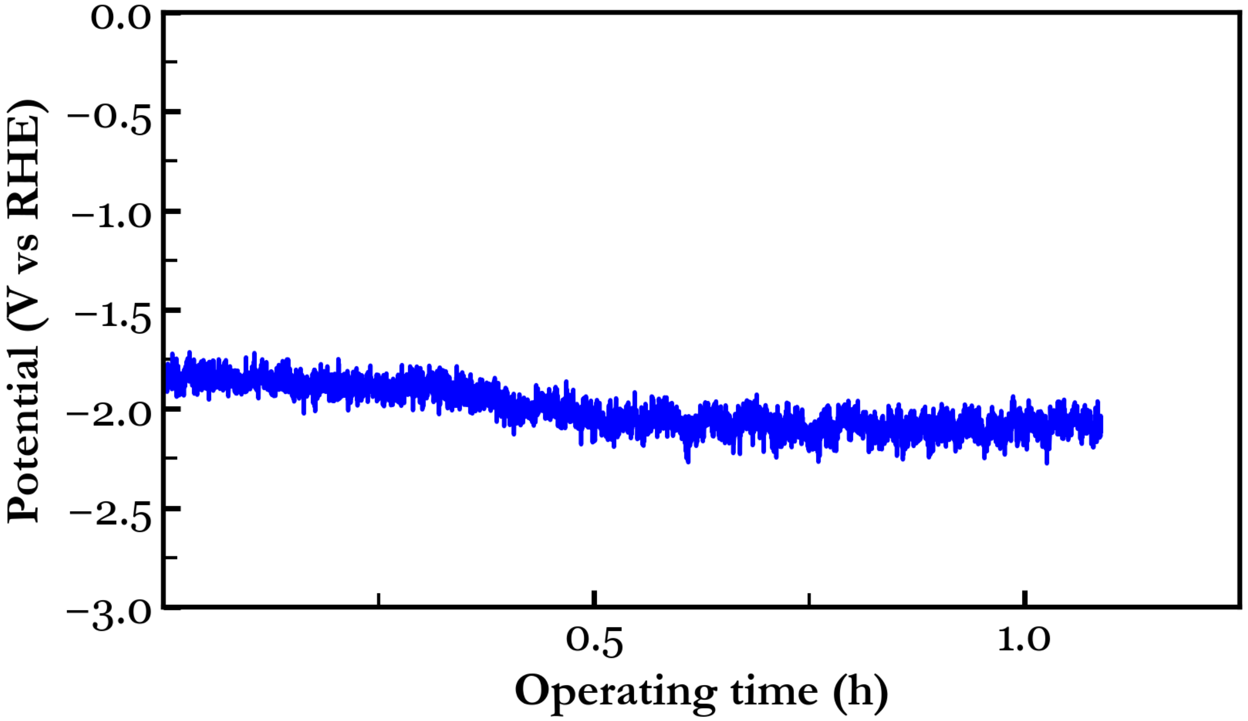

Figure S14. Uncompensated cathode potential (V vs RHE) of the copper GDE with a y-shaped current collector as a function of operating time at -200 mA·cm^{-2} in 1 M $KHCO_3$.

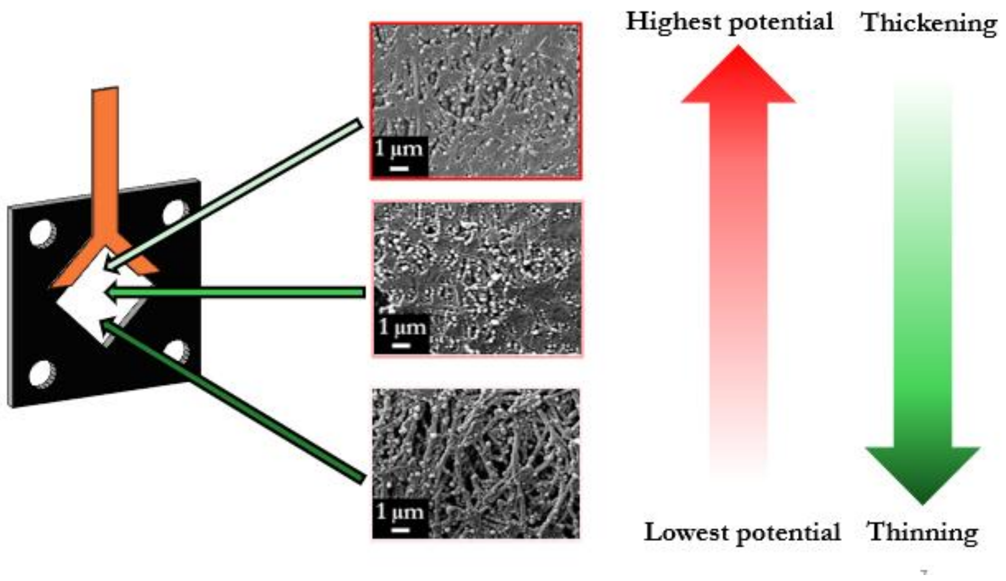

Figure S15. Postmortem SEM imaging at three different locations for the y-shaped current collector copper GDE after 60 minutes of operation.

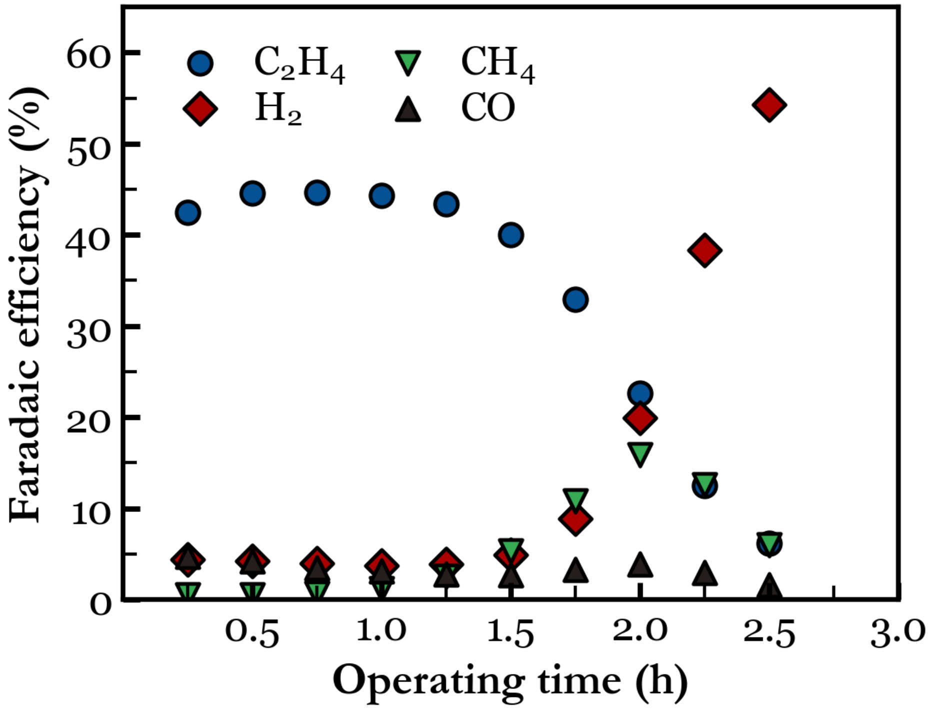

Figure S16. Faradaic efficiency profiles of gas products as a function of operating time at -200 mA·cm^{-2} using a bare copper GDE in 0.1 M $KHCO_3$.

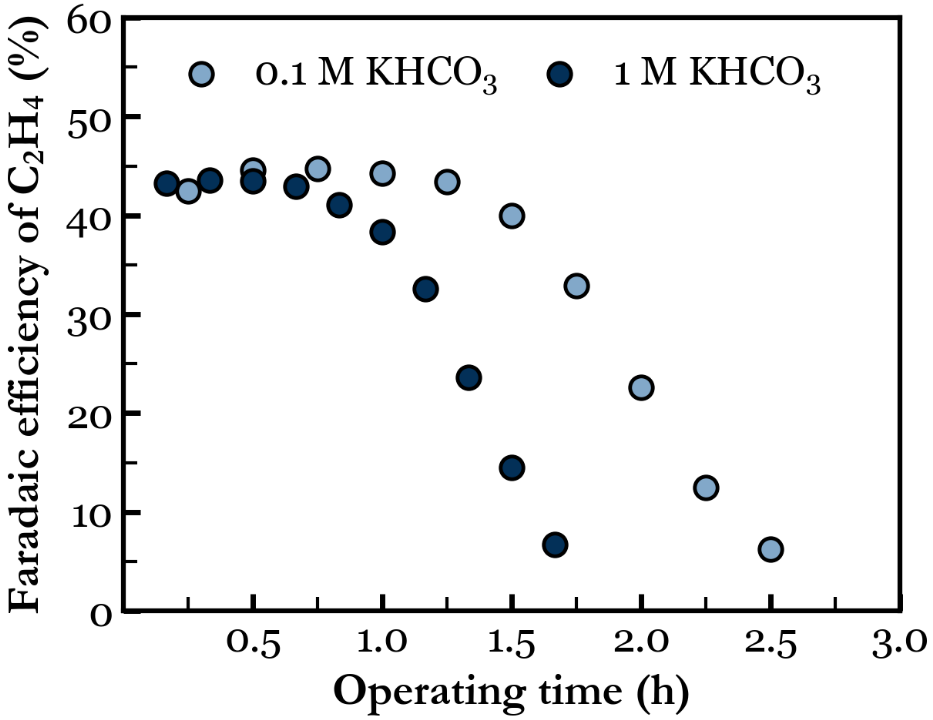

Figure S17. Buffer capacity comparison between the Faradaic efficiencies of ethylene as a function of time at -200 mA·cm^{-2} using a bare copper GDE in 0.1 M $KHCO_3$ and 1 M $KHCO_3$.

Results copper electrode with overlayers

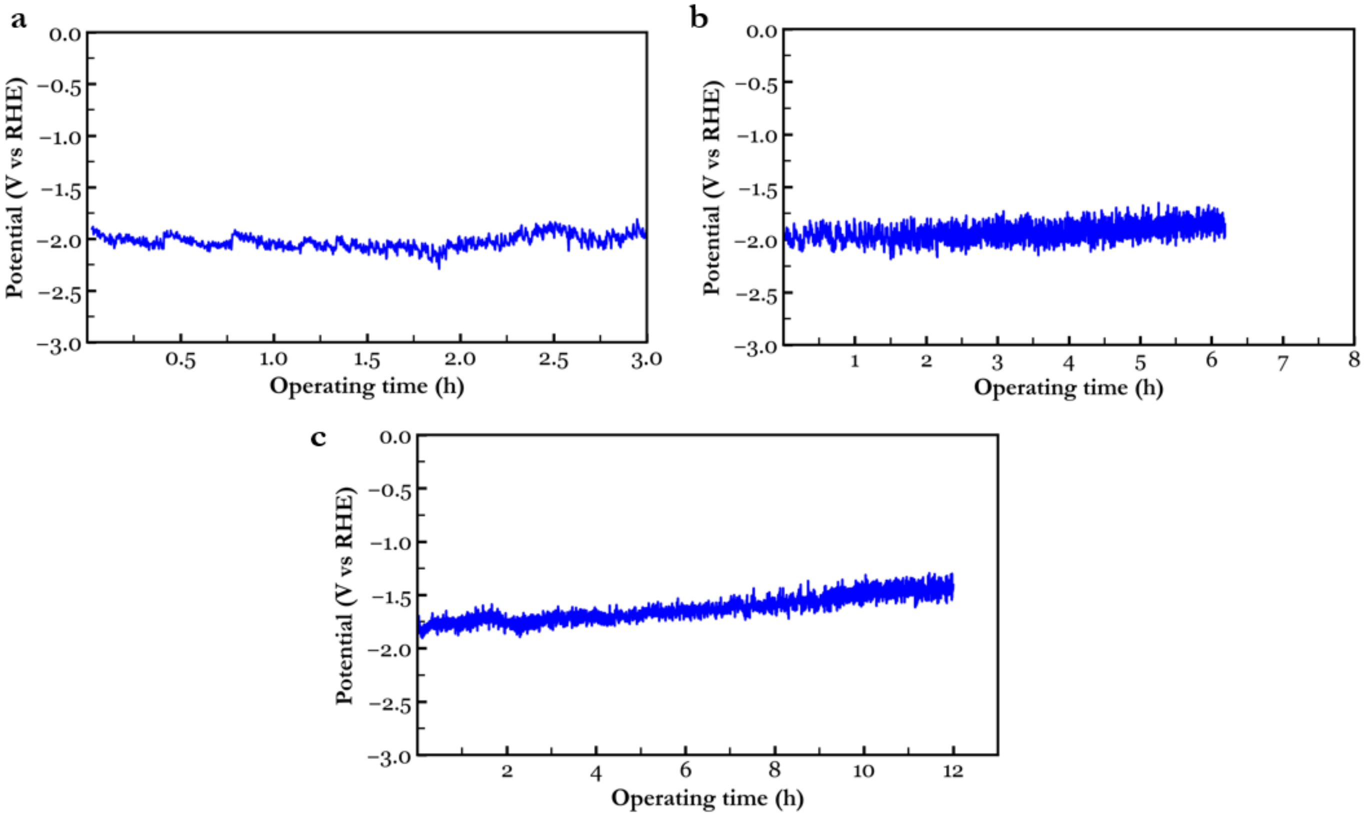

Figure S18. Uncompensated cathode potential as a function of operating time of the (a) bare copper, (b) Aquivion® coated copper, and (c) carbon NPs coated copper. All electrodes were subjected to -200 mA·cm^{-2} in 1 M $KHCO_3$.

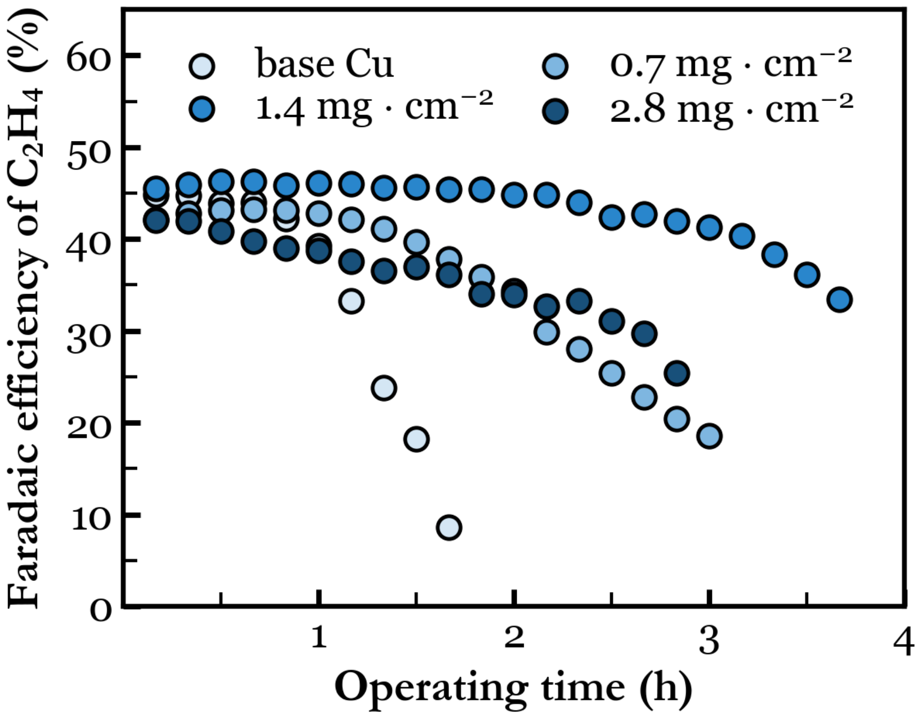

Figure S19. Faradaic efficiency of ethylene as a function of operating time for bare copper and copper with different areal loadings of Aquivion®.

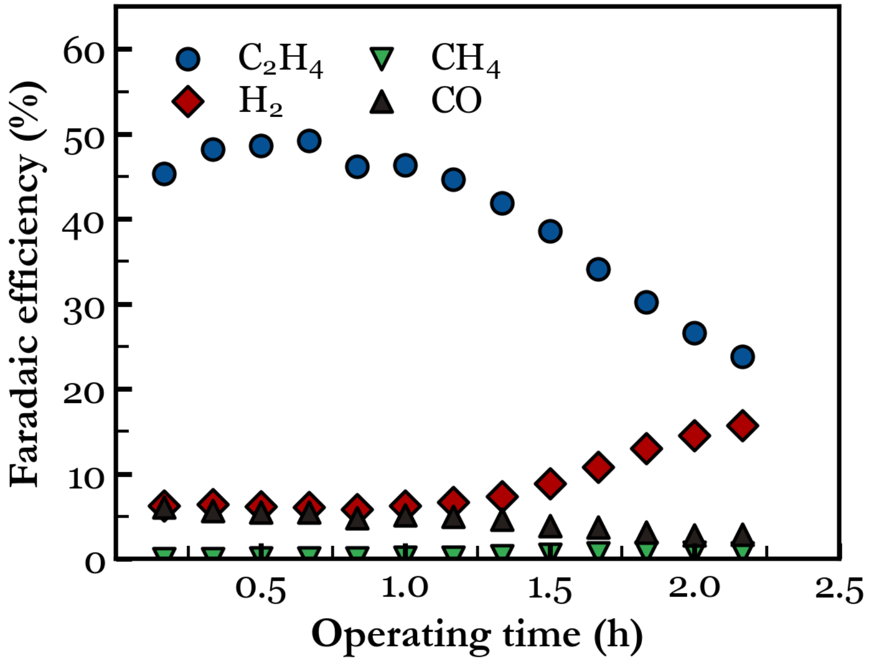

Figure S20. Faradaic efficiency profiles of gas products as a function of operating time for a sputtered copper electrode with 0.1 mg·cm^{-2} Sustainion® overlayer.

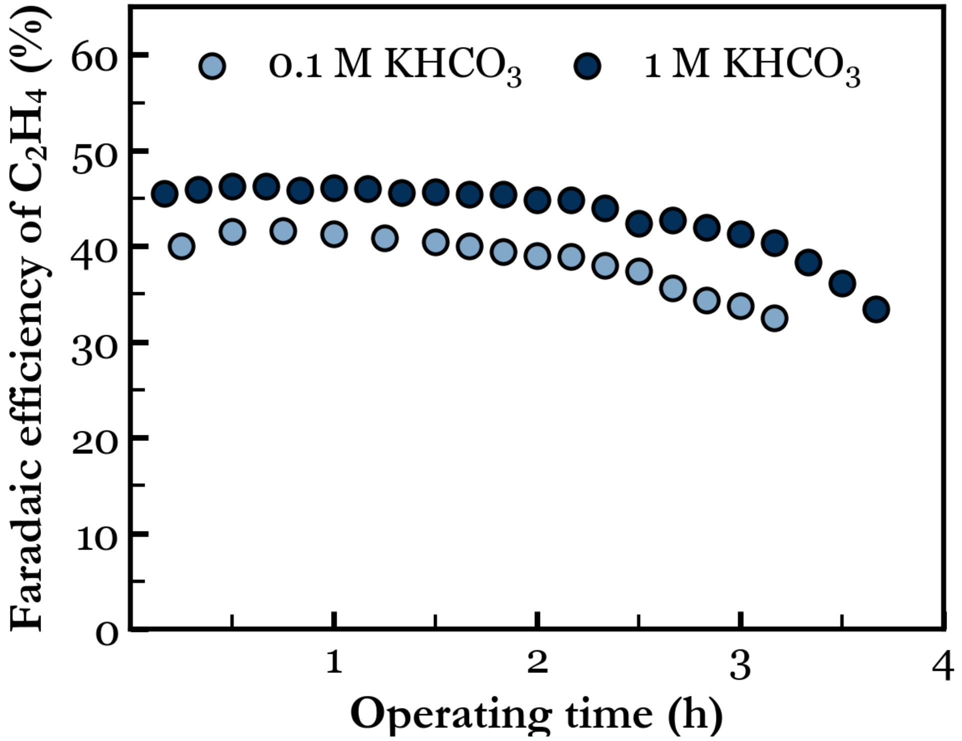

Figure S21. Faradaic efficiency of ethylene as a function of operating time at -200 mA·cm^{-2} in 0.1 M $KHCO_3$ and 1 M $KHCO_3$ using a sputtered copper electrode with an Aquivion® overlayer.

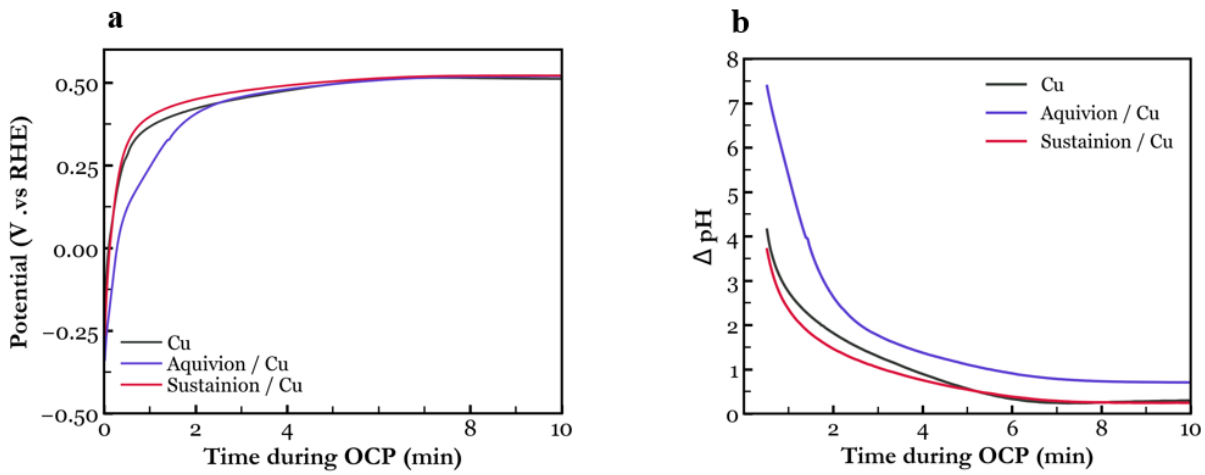

Figure S22. Open-circuit profile for different electrodes as a result of the local hydroxide-concentration as a function of time. (a) Open-circuit potential profiles of different electrodes after a period of electroreduction at -200 mA·cm^{-2}. (b) Difference in pH between bulk electrolyte and at the catalyst-electrolyte interface as described by the Nernst equation. ΔpH values are estimations as the initial drop in the open-circuit potentials is governed by capacitor discharge, not a change in local hydroxide-concentration.

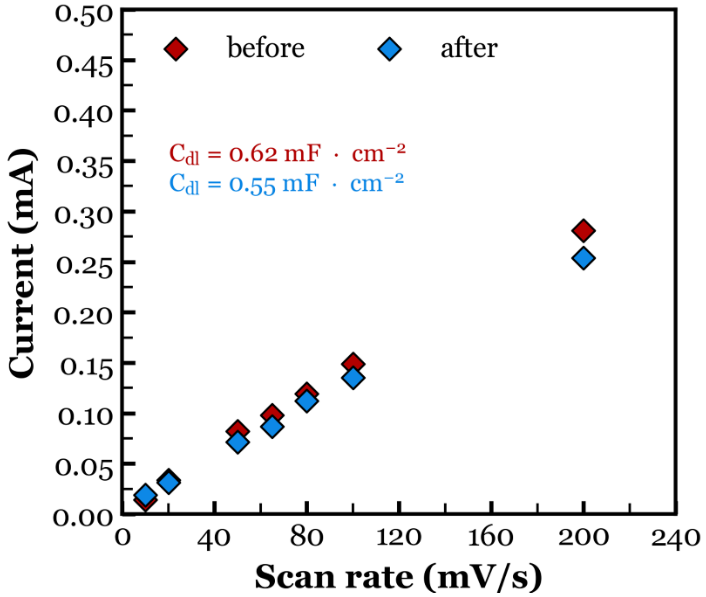

Figure S23. Average current as a function of scan rate before operation and after 80 minutes of operation of the sputtered copper electrode with an Aquivion® overlayer. Slope of curve is the capacitance, and its value is given in Table S3. Extracted capacitance values are included in the figure.

Table S3. Capacitance values before operation and after 80 minutes of operation of an Aquivion® coated copper GDE (see Fig. S23).

Experimental time	Capacitance value ($mF \cdot cm^{-2}$)
Before operation	0.62
After 80 minutes of operation	0.55

Table S4. ICP-OES data of Aquivion® coated copper sample at 80 and 375 minutes of operation. Bracketed numbers represent the wavelength used for the ppm determination.

Sample name	Sample Location	Cu (ppm) (213, 598 nm)	Cu (ppm) (324, 754 nm)	Cu (ppm) (327, 395 nm)	Average (ppm)
Aquivion®/Cu after 80 min operation	Perimeter	6.05	5.69	5.69	5.81
	Center	6.08	5.75	5.74	5.86
Aquivion®/Cu after 375 minutes of operation	Perimeter	5.92	5.53	5.53	5.66
	Center	5.92	5.56	5.54	5.67

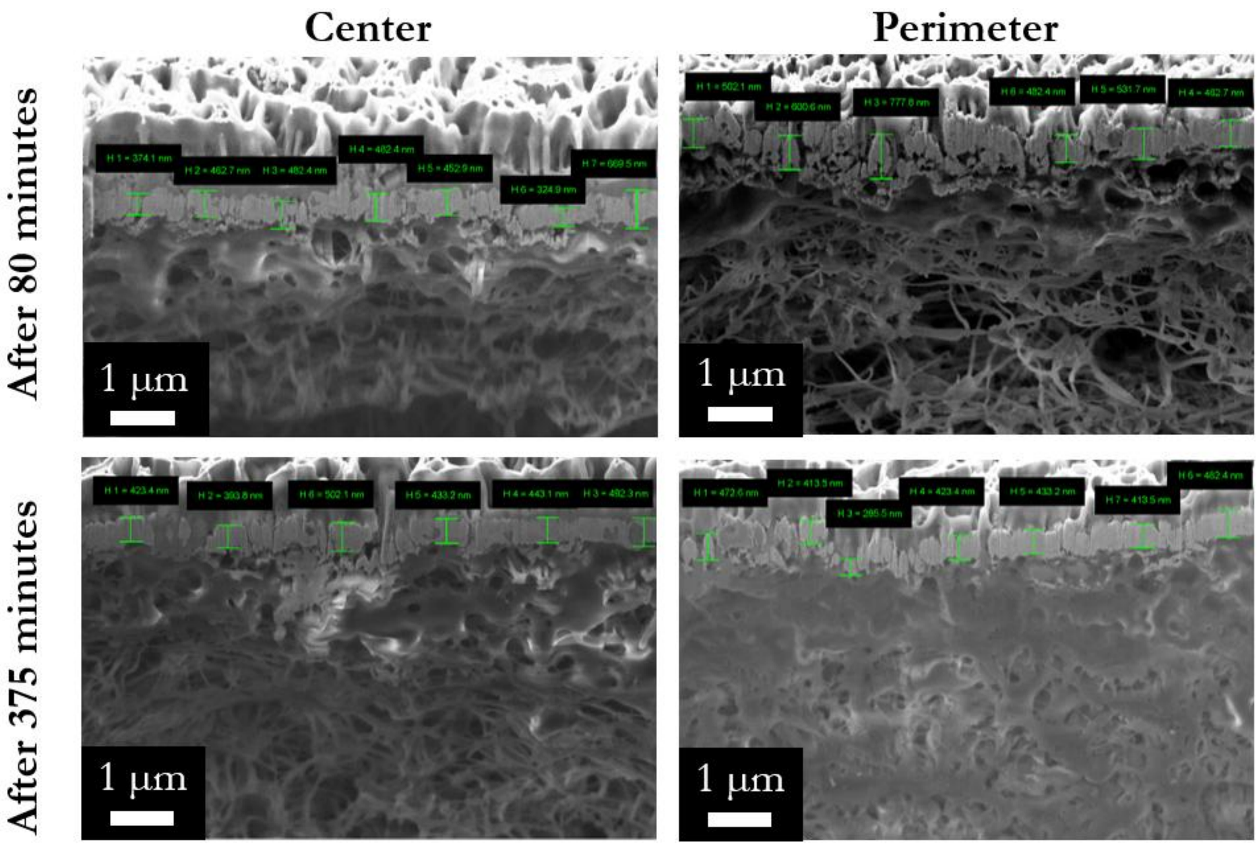

Figure S24. FIB analysis revealing the cross-sectional morphology of the center and perimeter of a sputtered copper electrode with an Aquivion® overlayer after 80 minutes and 375 minutes of operation. Green bars indicate where thickness measurements were taken.

Table S5. ICP-OES data of carbon NPs coated copper sample at 80 and 465 minutes of operation. Bracketed numbers represent the wavelength used for the ppm determination.

Sample name	Perimeter or center	Cu (ppm) (213, 598 nm)	Cu (ppm) (324, 754 nm)	Cu (ppm) (327, 395 nm)	Average (ppm)
Carbon/Cu after 80 min operation	Perimeter	5.30	5.76	5.72	5.59
	Center	4.79	5.15	5.11	5.02
Carbon/Cu after 465 minutes of operation	Perimeter	5.15	5.78	5.73	5.55
	Center	5.82	5.47	5.48	5.59

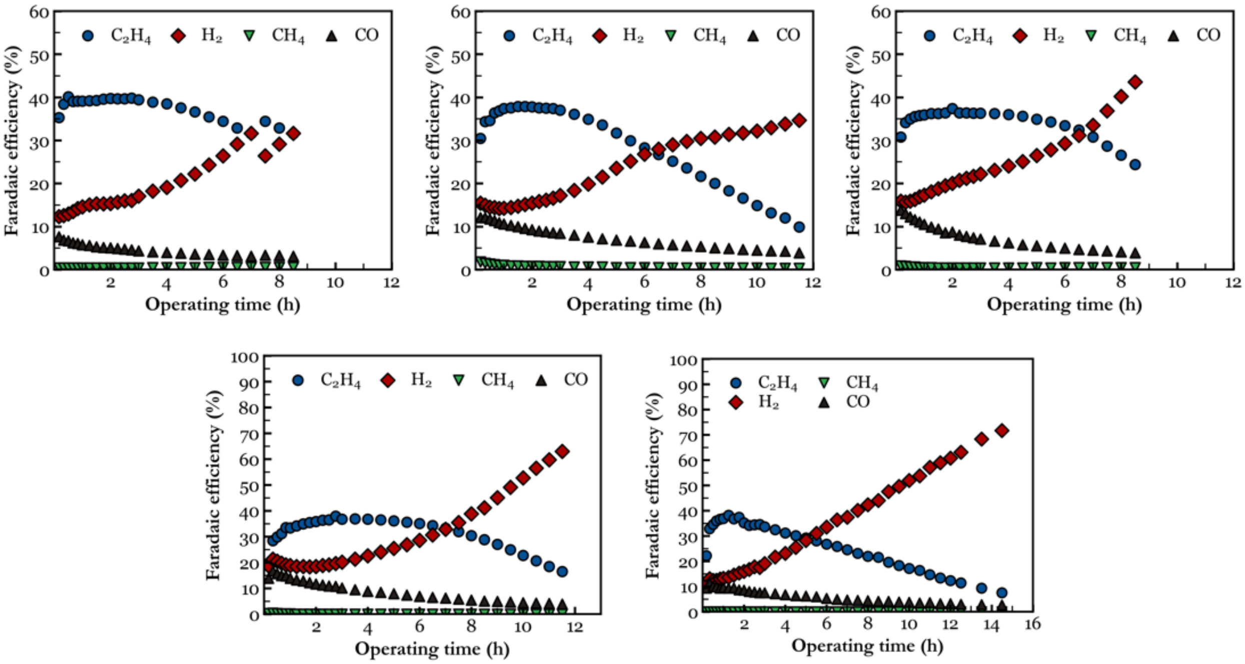

Figure S25. The Faradaic efficiency profiles of gas products during five independent CO_2 electroreduction experimentson a carbon NPs coated copper GDE in 1 M $KHCO_3$ at a current density of -200 mA·cm^{-2}. The two bottom experiments include hydrogen from both the gas channel and from the catholyte headspace, which explains why explains why the Faradaic efficiency values for hydrogen are significantly higher compared to the others (see Figure S32).

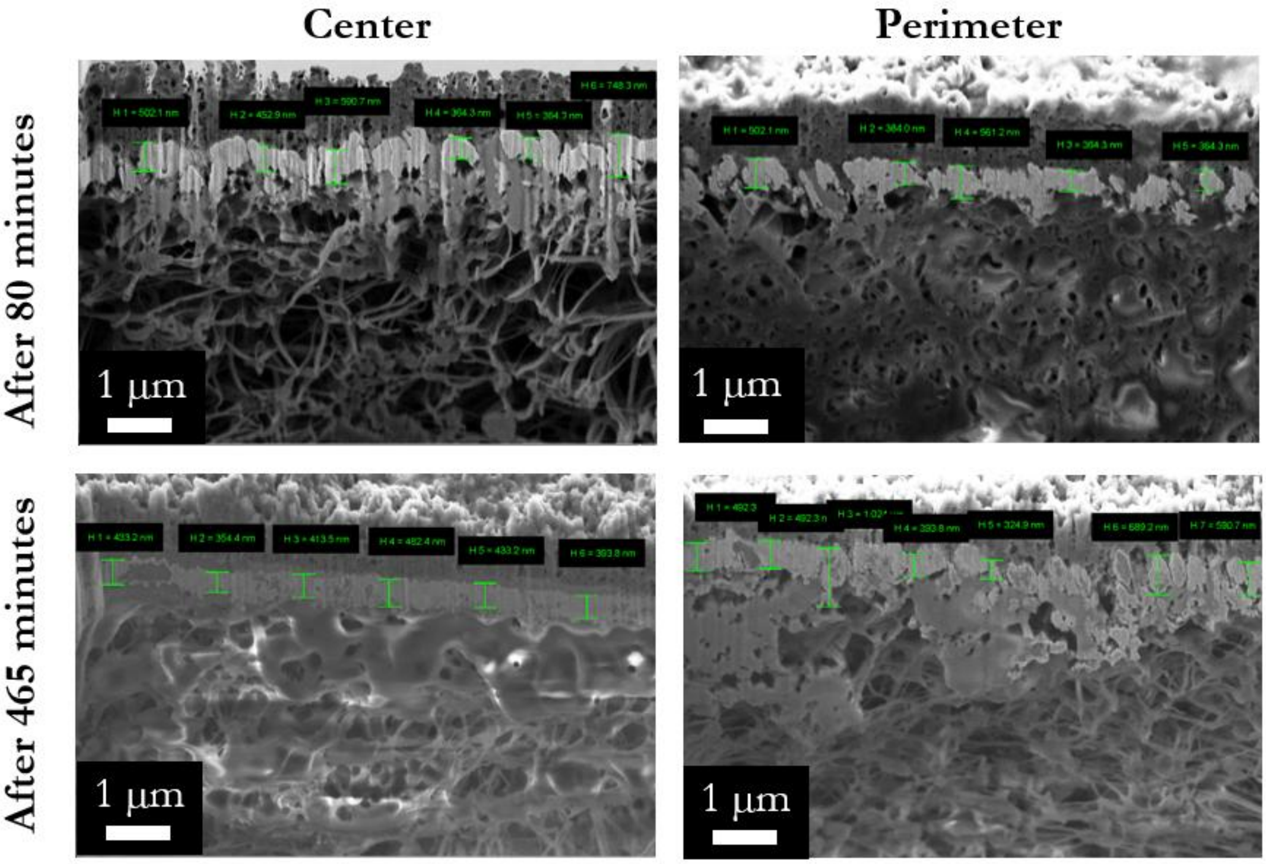

Center
Perimeter
After 80 minutes
After 465 minutes
1 μm
1 μm
1 μm
1 μm

Figure S26. FIB analysis revealing the cross-sectional morphology of the center and perimeter of the carbon NPs coated copper GDE at 80 minutes and after 465 minutes of operation. Green bars indicate where thickness measurements were taken.

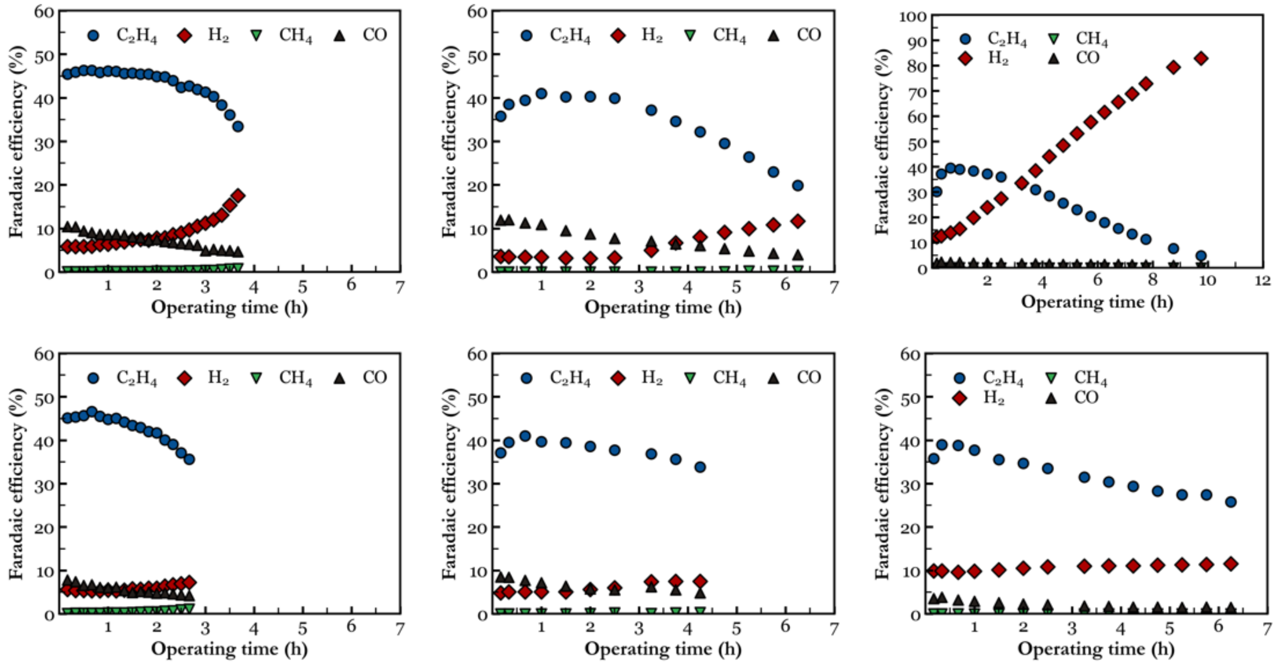

Figure S27. The Faradaic efficiency profiles of gas products during six independent CO_2 electroreduction experiments on a sputtered copper electrode with an Aquivion® overlayer in 1 M $KHCO_3$ at a current density of –200 mA·cm^{-2}. The most upper right experiment takes hydrogen leaving through the catholyte into consideration (see Figure S32).

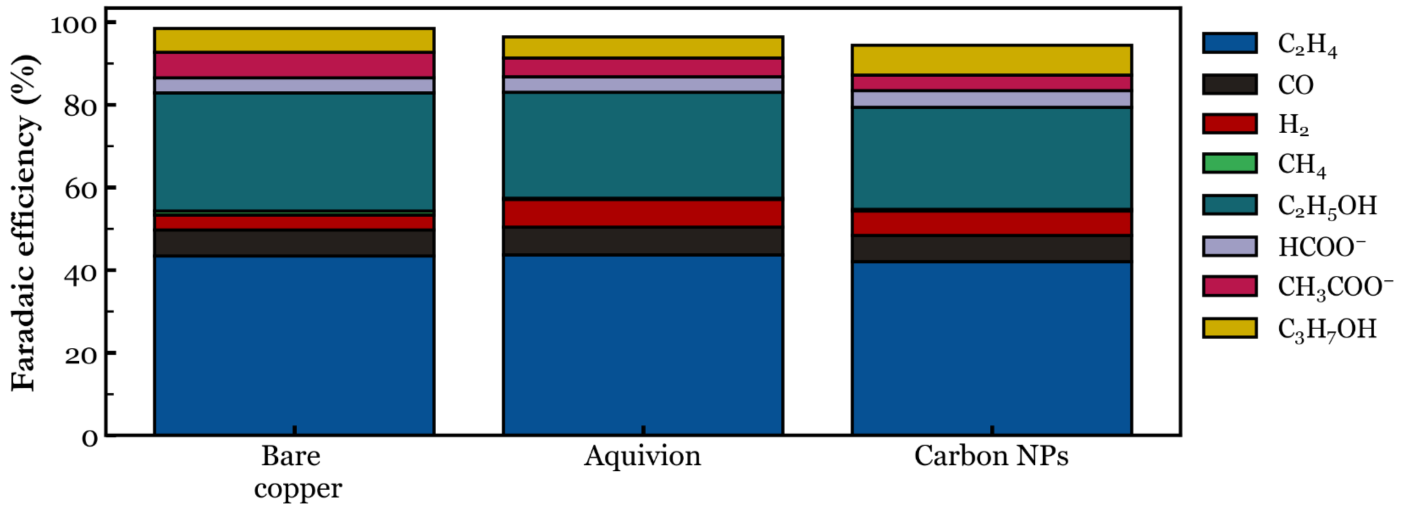
Faradaic efficiency (%)
100
80
60
40
20
0
Bare copper
Aquivion
Carbon NPs
C_2H_4
CO
H_2
CH_4
C_2H_5OH
$HCOO^-$
CH_3COO^-
C_3H_7OH

Figure S28. Full product distribution of different GDEs. The bare copper electrode (0.5 h), Aquivion® overlayer (3 h), and carbon nanoparticles overlayer (3 h).

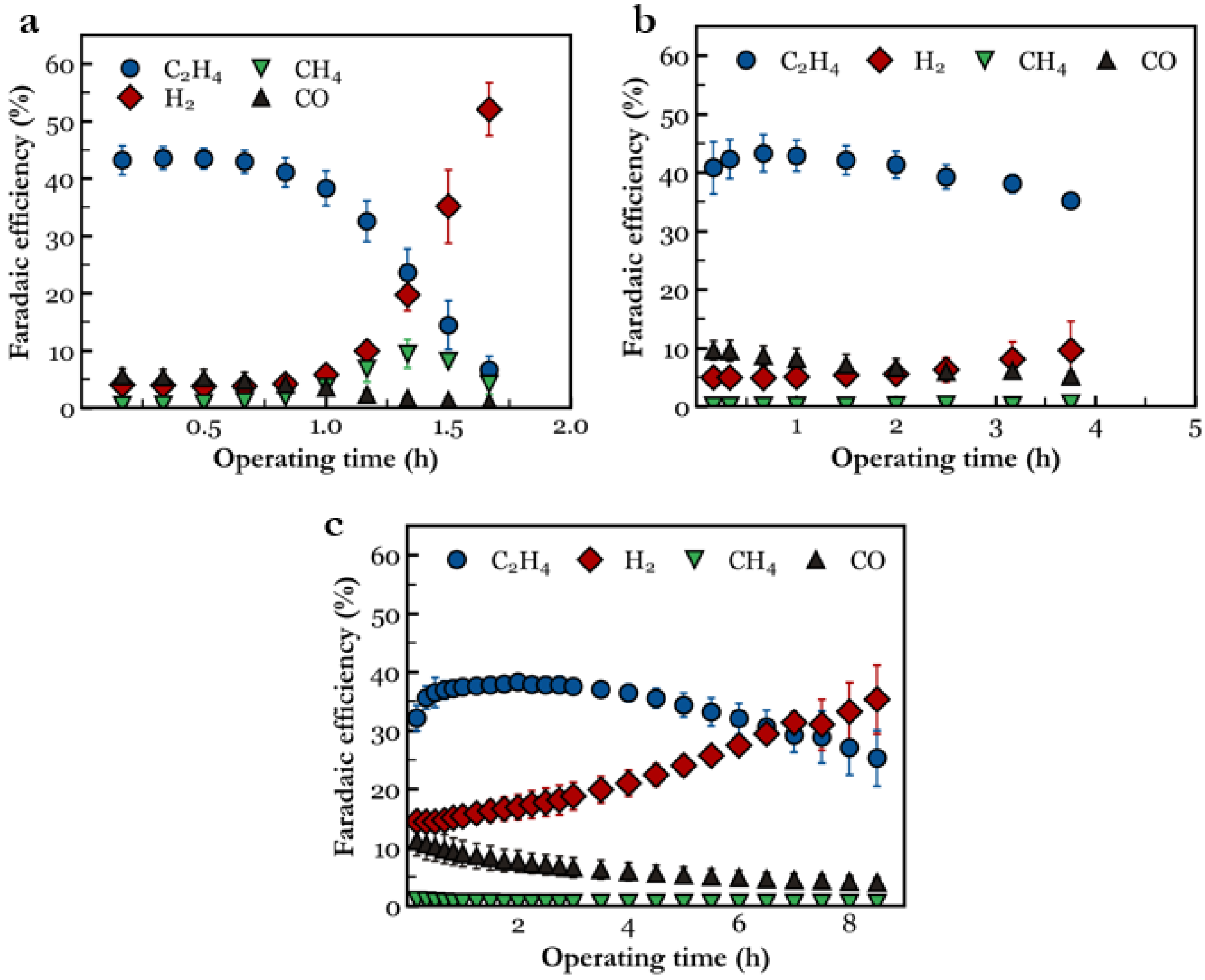

Figure S29. Average Faradaic efficiency profiles of gas products as a function of time with error bars. Error bars represent standard deviation of at least three independent experiments. (a) Bare copper (n = 4). (b) Aquivion® coated copper GDE (n = 3). (c) Carbon NPs coated copper GDE (n = 3).

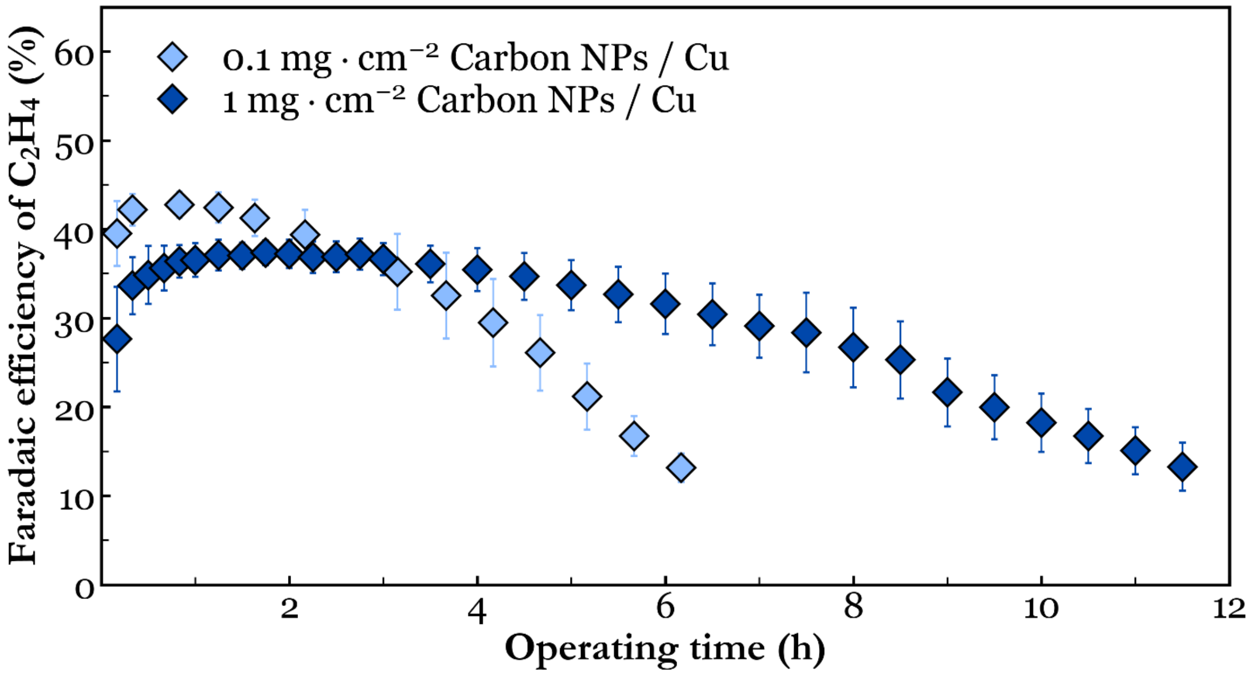

Figure S30. Faradaic efficiency profile of ethylene as a function of operating time for different loadings of carbon NPs on top of a sputtered copper catalyst. Error bars represent standard deviation of at least three independent experiments.

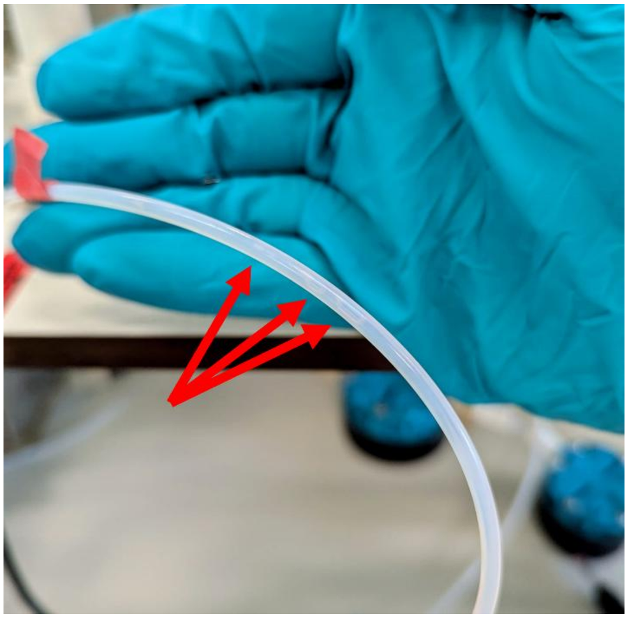

Figure S31. Hydrogen bubble formation in catholyte tubes near the end of the experiment when hydrogen takes over as the primary observed product.

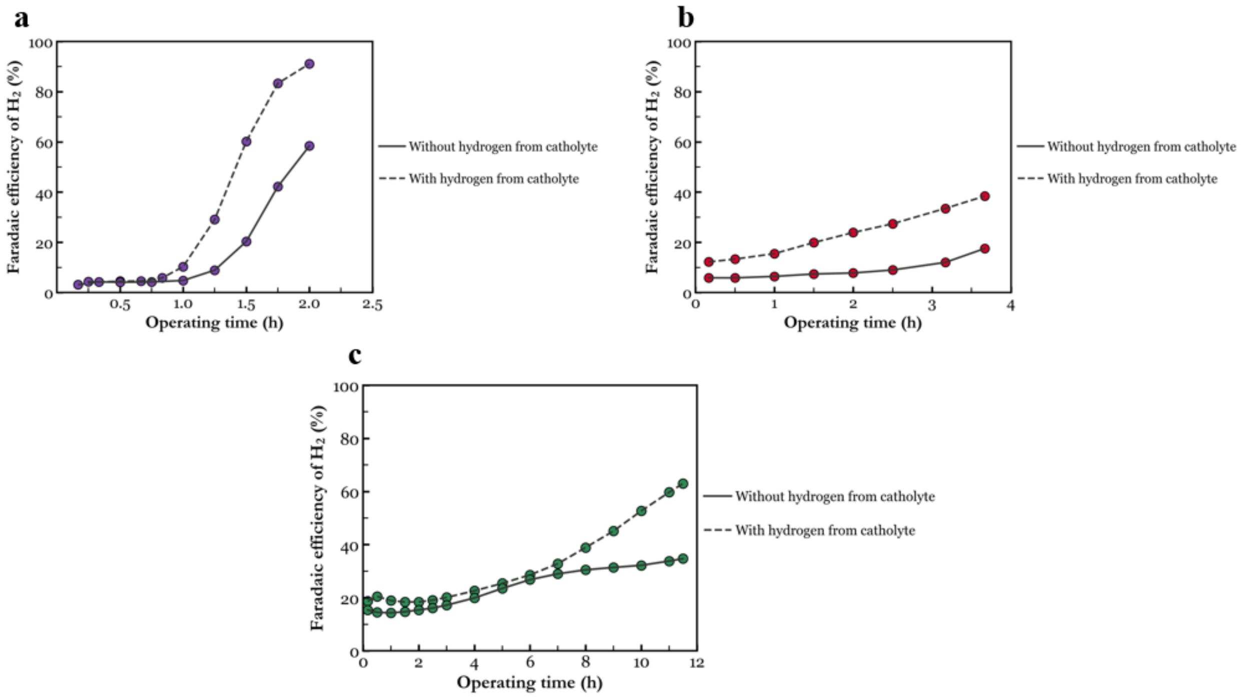

Figure S32. Faradaic efficiency profiles of hydrogen with and without taking the hydrogen leaving the flow cell through the electrolyte into consideration. (a) Bare copper electrode. (b) Aquivion® coated copper electrode. (c) Carbon NPs coated copper electrode.

a

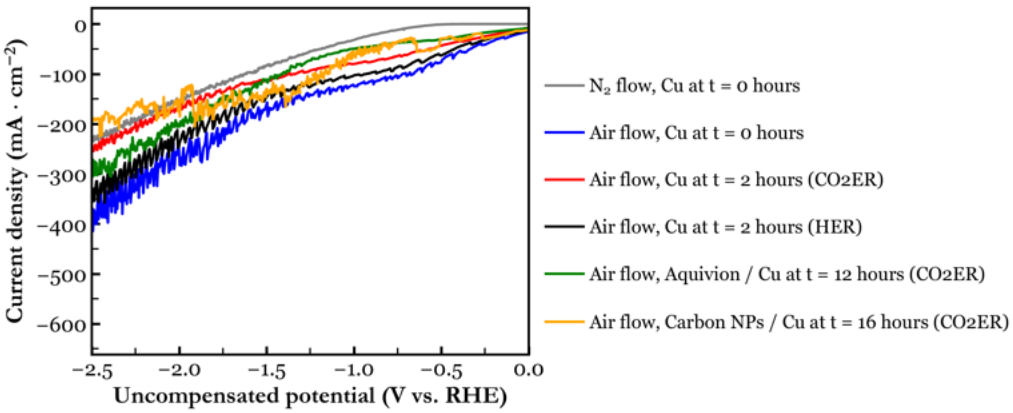
Current density (mA · cm⁻²)
0
−100
−200
−300
−400
−500
−600
−2.5
−2.0
−1.5
−1.0
−0.5
0.0
Uncompensated potential (V vs. RHE)
N_2 flow, Cu at t = 0 hours
Air flow, Cu at t = 0 hours
Air flow, Cu at t = 2 hours (CO2ER)
Air flow, Cu at t = 2 hours (HER)
Air flow, Aquivion / Cu at t = 12 hours (CO2ER)
Air flow, Carbon NPs / Cu at t = 16 hours (CO2ER)

b

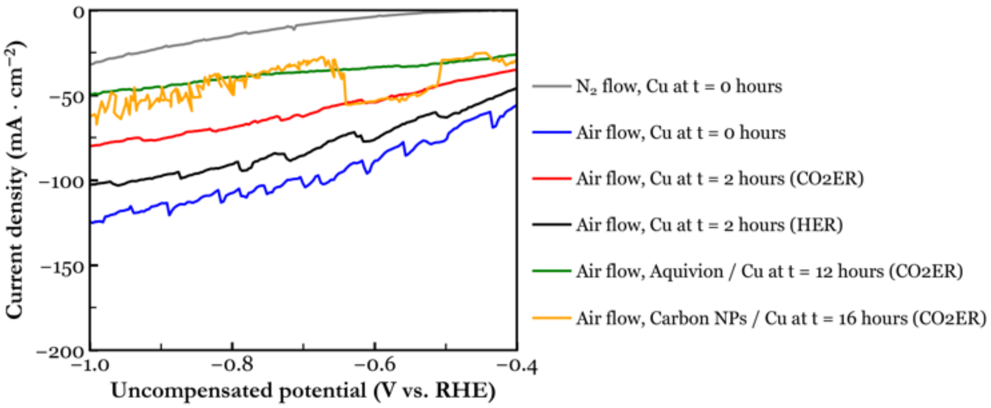
Current density (mA · cm⁻²)
0
−50
−100
−150
−200
−1.0
−0.8
−0.6
−0.4
Uncompensated potential (V vs. RHE)
N_2 flow, Cu at t = 0 hours
Air flow, Cu at t = 0 hours
Air flow, Cu at t = 2 hours (CO2ER)
Air flow, Cu at t = 2 hours (HER)
Air flow, Aquivion / Cu at t = 12 hours (CO2ER)
Air flow, Carbon NPs / Cu at t = 16 hours (CO2ER)

Figure S33. Results of oxygen reduction linear sweep voltammetry for various electrode samples in a flow cell configuration using N_2 or air flow in the gas channel of the electrochemical cell. In air conditions the oxygen reduction reaction occurs at more positive potentials before hydrogen evolution begins. (a) shows the potential range from 0 to -2.5 V vs RHE. (b) shows the potential range from -0.4 to -1.0 V vs RHE. Note that the potential has not been compensated for the ohmic drop between the working and reference electrode (distance = 0.75 cm).

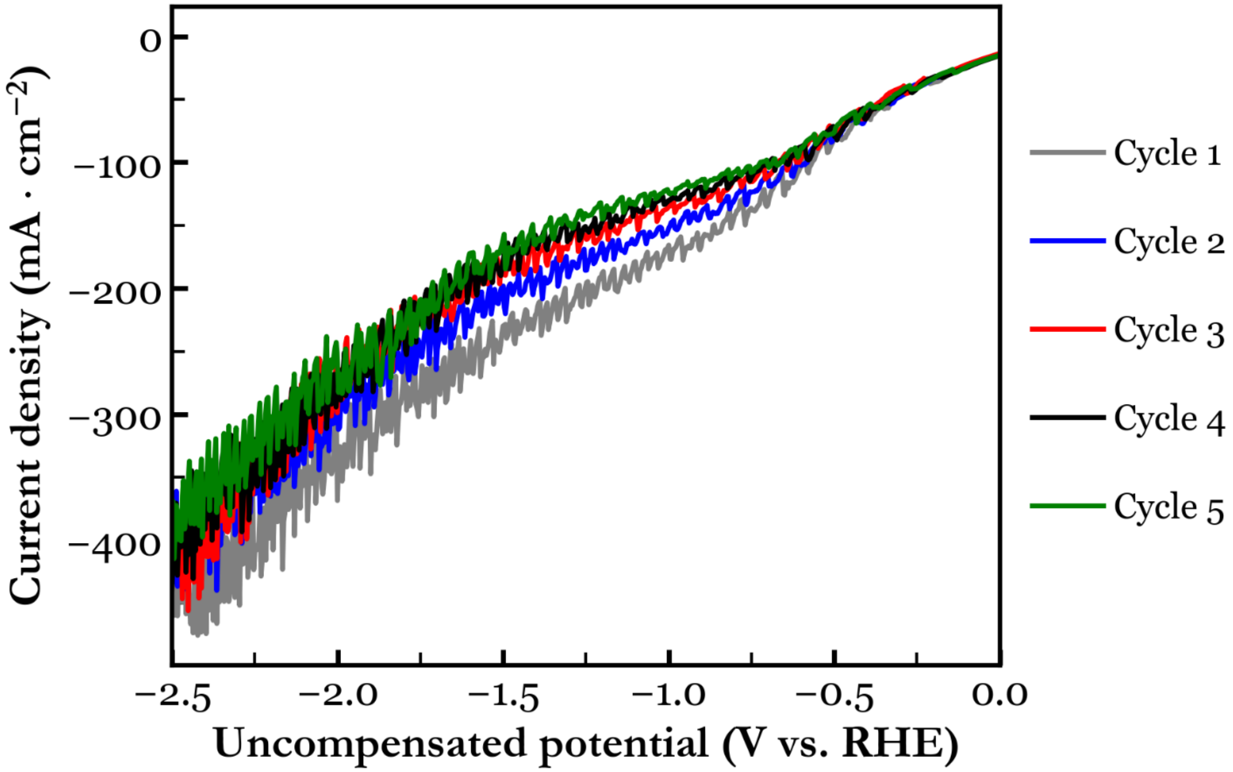

Figure S34. Multiple cycles were needed before the curves within the oxygen reduction reaction potential range started to overlap.

Table S6. Approximate oxygen reduction reaction (ORR) limiting current densities observed for different gas flow and catalyst architectures via linear sweep voltammetry (LSV).

Gas Flow	Operating Time and Conditions prior to ORR LSV	Catalyst Layer	ORR Limiting Current Density ($j_{ORR,lim}$)
N_2	0 hours	500 nm Cu	0
Air	0 hours	500 nm Cu	-100 to -120
Air	2 hours, N_2 gas flow	500 nm Cu	-90 to -100
Air	2 hours, CO_2 gas flow	500 nm Cu	-75 to -90

Air	12 hours, CO_2 gas flow	500 nm Cu + Aquivion	-30 to -50
Air	16 hours, CO_2 gas flow	500 nm Cu + 1 mg cm^{-2} carbon nanoparticles	-30 to -50

Results MEA test

Table S7. Flow cell works reporting their current collector dimensions and stability.

Operating current density (mA · cm^{-2})	Maximum distance from current collector to copper catalyst (cm)	Stability (h)	Reference
300	1 (tape)	45	[3]
150	1 (tape)	10	[4]
663	0.7 (tape)	30	[5]
100	1.1 (tape)	1.25	[6]
150	1 (tape)	0.7	[7]

Table S8. MEA cells reporting their current collector dimensions and stability.

Operating current density (mA · cm^{-2})	Maximum distance from current collector to copper catalyst (cm)	Stability (h)	Reference
480	0.05	3.50	[8]
150	0.05	65	[9]
200	0.05	24	[10]

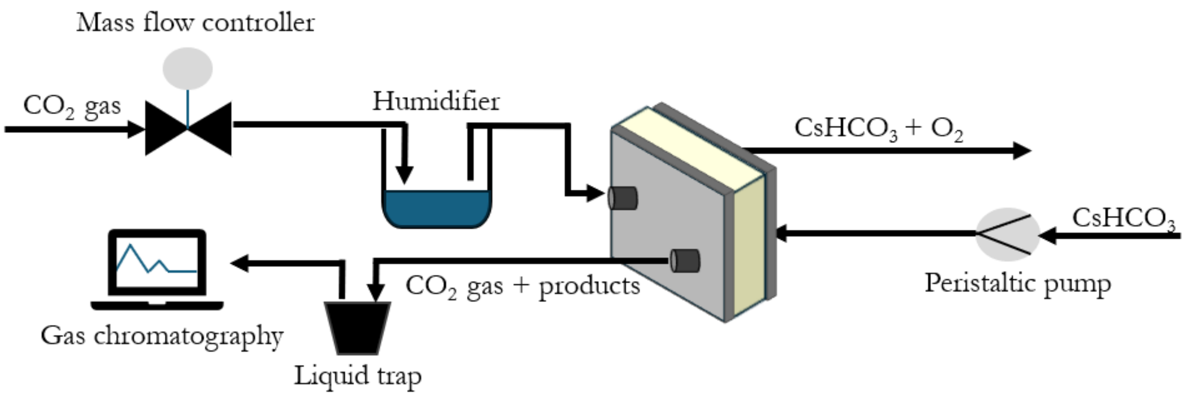

Figure S35. MEA cell experimental setup used to perform experiments.

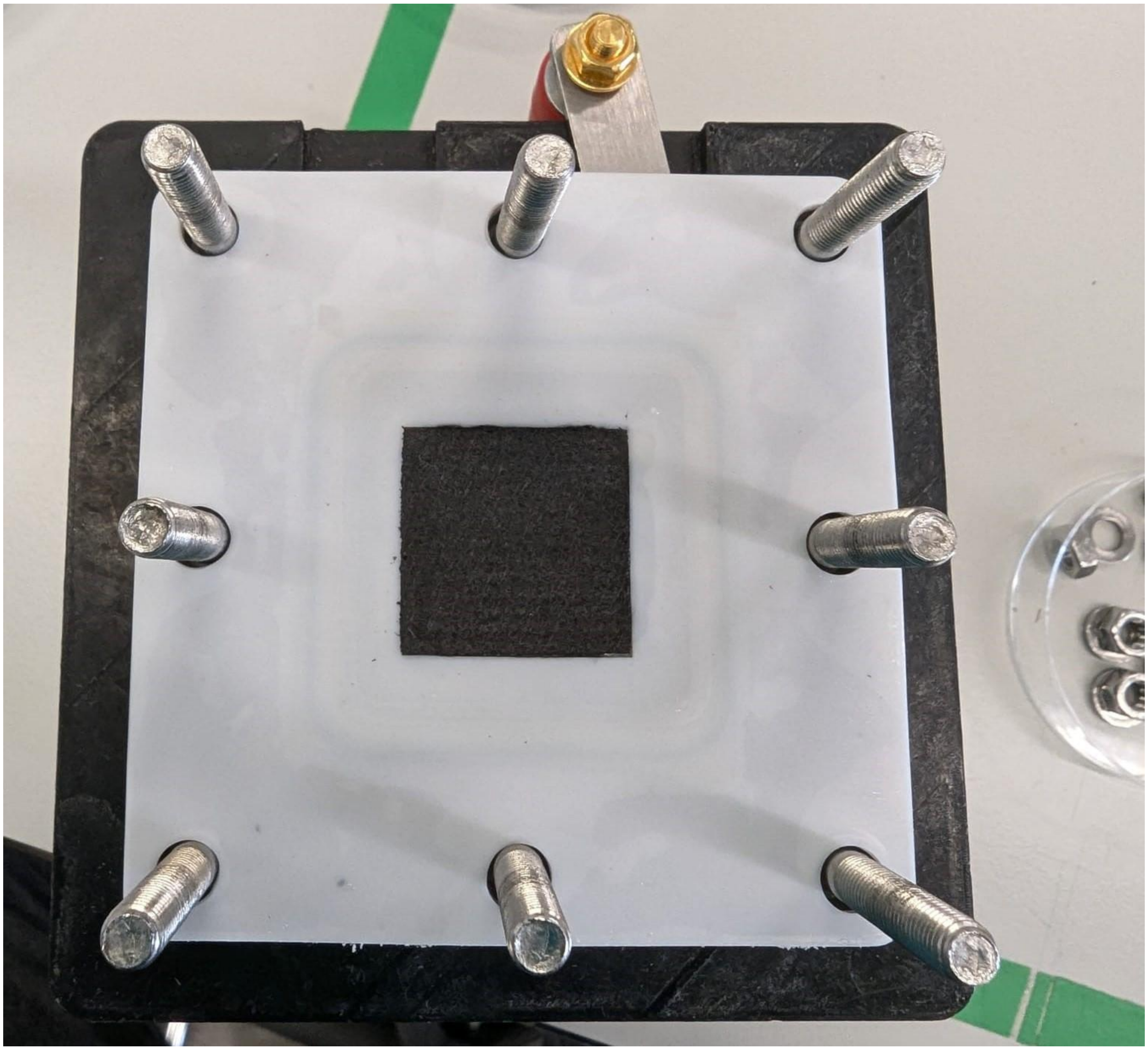

Figure S36. Image showing absence of salt particles at back of copper GDE post operation.

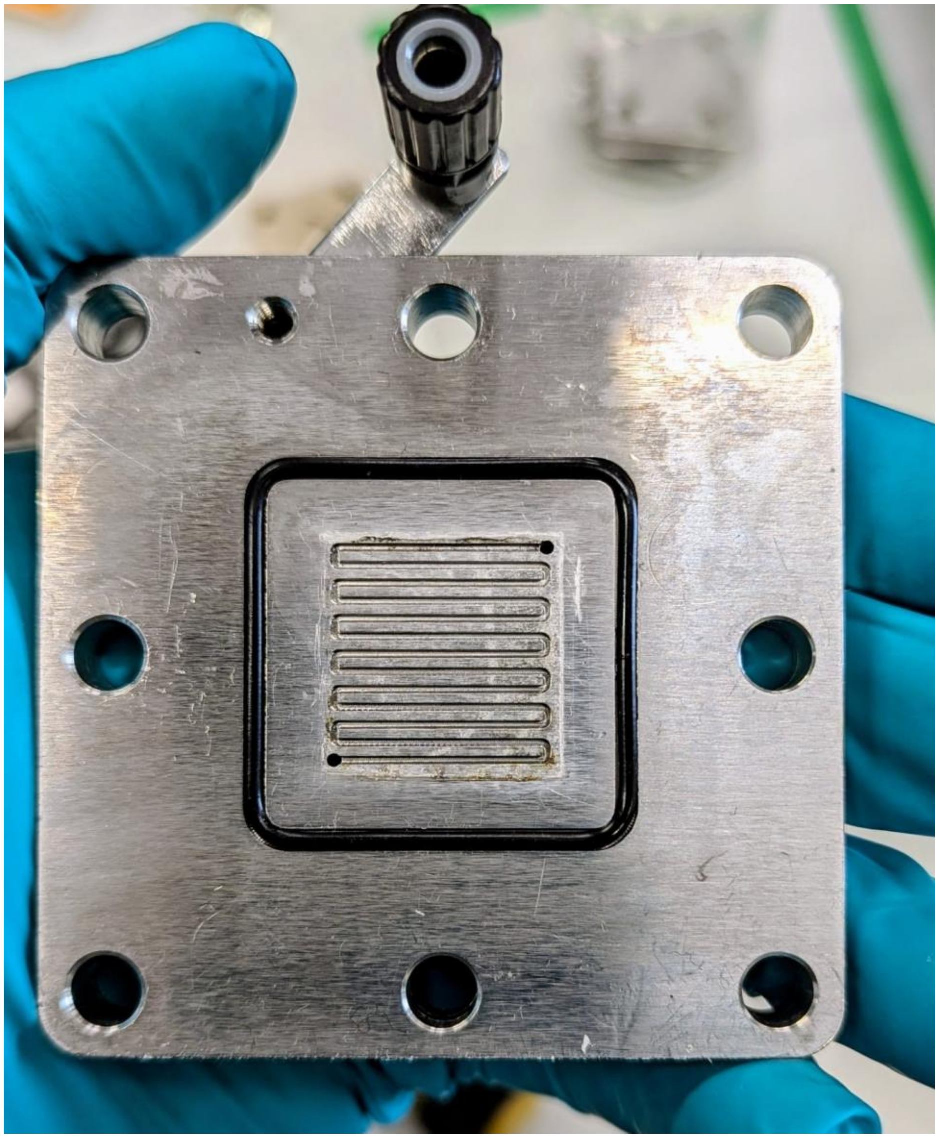

Figure S37. Image showing the cathode serpentine flow field pattern. No salt particles were found after the MEA stability test.

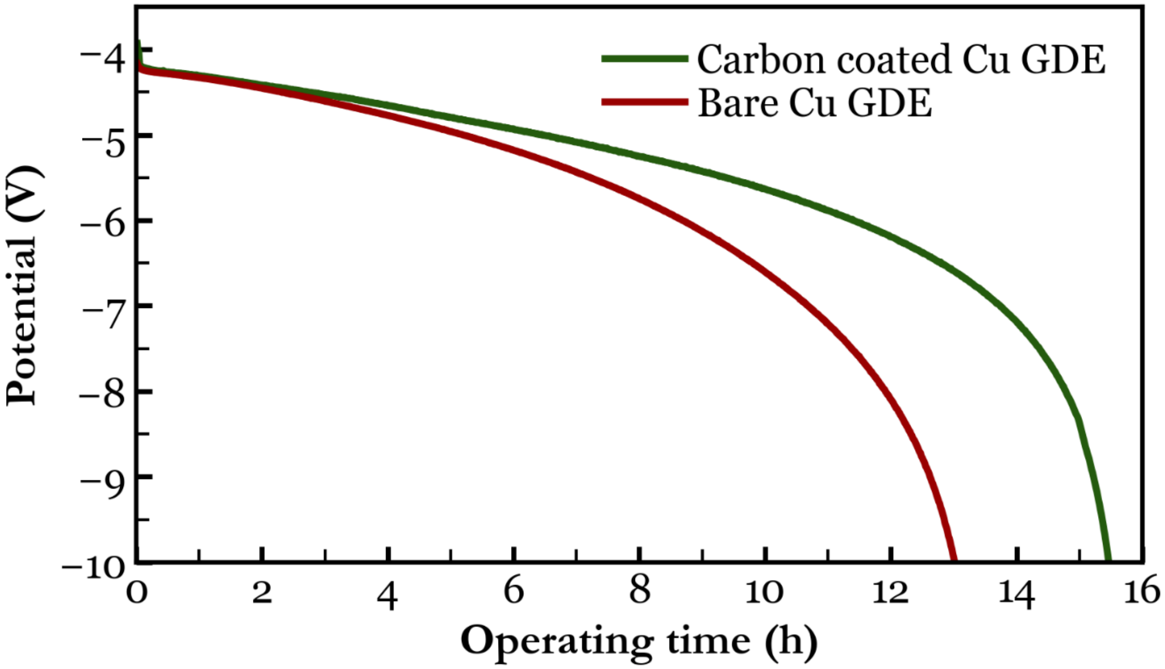

Figure S38. Potential as a function of operating time during MEA stability test.

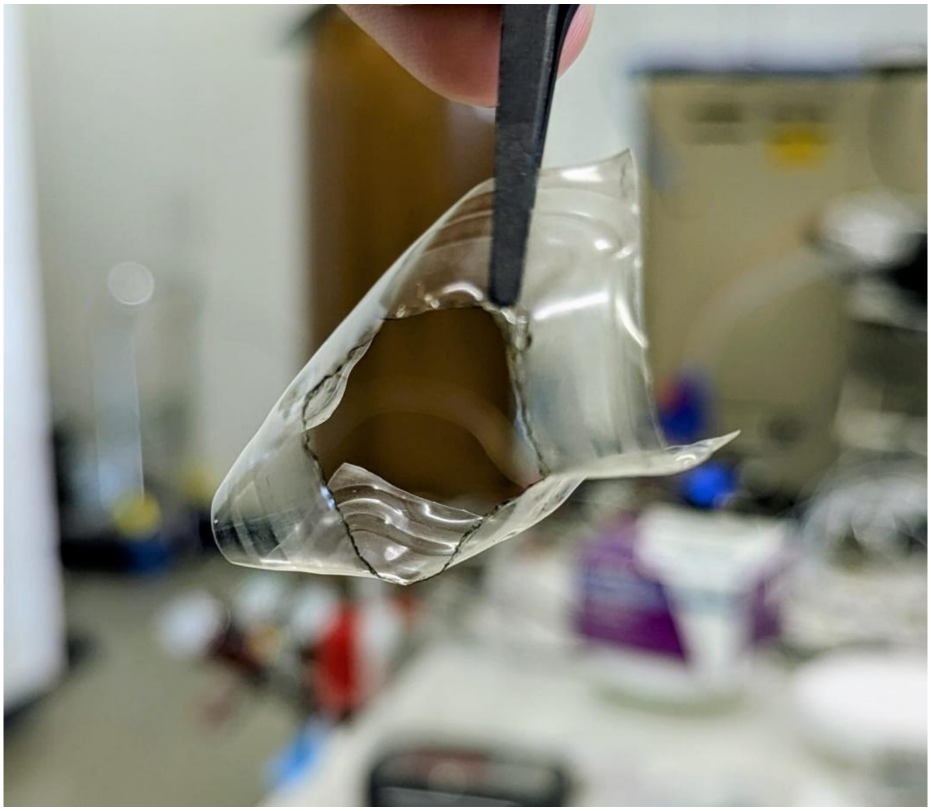

Figure S39. PiperION® membrane post stability test showing cavity. It is hypothesized to occur because of ethanol-induced dissolution.

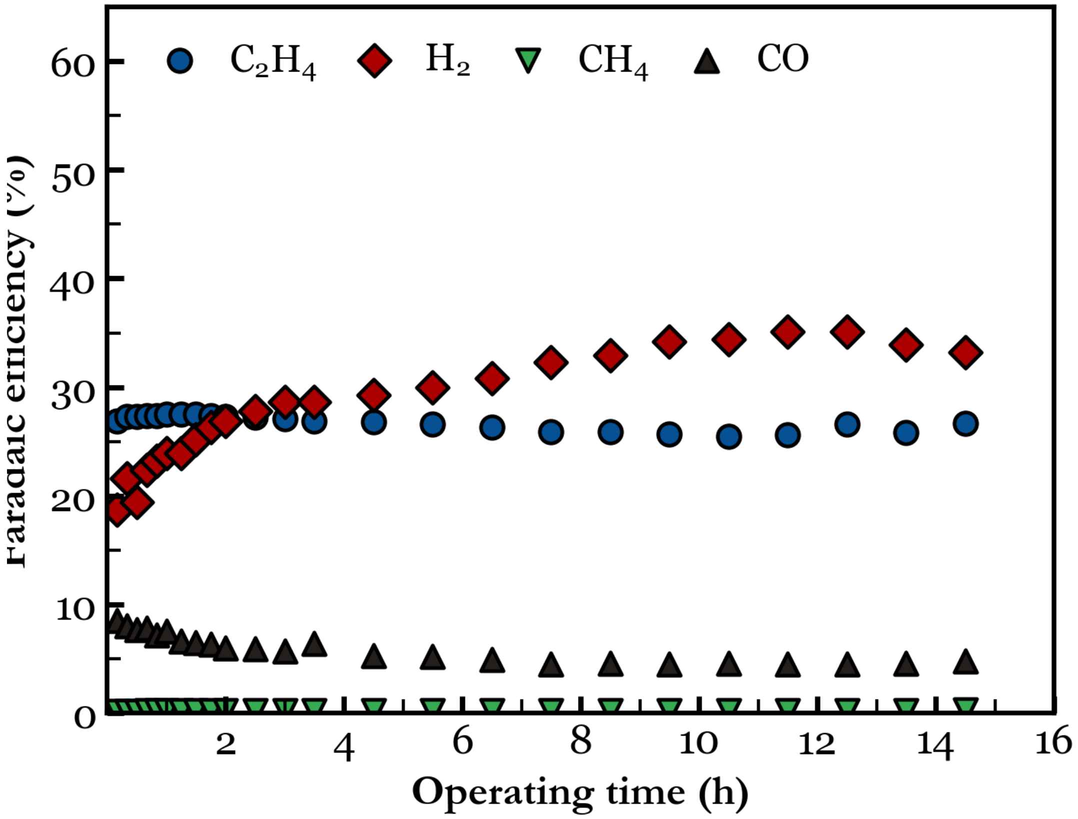

Figure S40. FE profiles of gas product produced during stability test in an MEA cell at a current density of -200 mA·cm^{-2} with a carbon NPs coated copper GDE (Sigracet 39 BB). Immediate rise of hydrogen selectivity was attributed to the dissolvement of the PiperION® membrane. Lower FE production due to the required prior wetting of PiperION® membrane with 1 M $CsHCO_3$.

References

1. Iglesias Van Montfort, H.-P. *et al.* An Advanced Guide to Assembly and Operation of CO_2 Electrolyzers. *ACS Energy Lett.* **8**, 4156–4161 (2023).
2. Baumgartner, L. M., Koopman, C. I., Forner-Cuenca, A. & Vermaas, D. A. Narrow Pressure Stability Window of Gas Diffusion Electrodes Limits the Scale-Up of CO_2 Electrolyzers. *ACS Sustain. Chem. Eng.* **10**, 4683–4693 (2022).
3. Niu, Z.-Z. *et al.* Hierarchical Copper with Inherent Hydrophobicity Mitigates Electrode Flooding for High-Rate CO_2 Electroreduction to Multicarbon Products. *J. Am. Chem. Soc.* **143**, 8011–8021 (2021).
4. Liu, W. *et al.* Electrochemical CO2 reduction to ethylene by ultrathin CuO nanoplate arrays. *Nat. Commun.* **13**, 1877 (2022).
5. Ma, Z. *et al.* CO2 electroreduction to multicarbon products in strongly acidic electrolyte via synergistically modulating the local microenvironment. *Nat. Commun.* **13**, 7596 (2022).
6. Kok, J., De Ruiter, J., Van Der Stam, W. & Burdyny, T. Interrogation of Oxidative Pulsed Methods for the Stabilization of Copper Electrodes for CO_2 Electrolysis. *J. Am. Chem. Soc.* **146**, 19509–19520 (2024).
7. Nguyen, T. N. *et al.* Catalyst Regeneration via Chemical Oxidation Enables Long-Term Electrochemical Carbon Dioxide Reduction. *J. Am. Chem. Soc.* **144**, 13254–13265 (2022).
8. Zhang, G. *et al.* Efficient CO2 electroreduction on facet-selective copper films with high conversion rate. *Nat. Commun.* **12**, 5745 (2021).
9. Papangelakis, P. *et al.* Carbon-Efficient CO_2 Electrolysis to Ethylene with Nanoporous Hydrophobic Copper. *Adv. Energy Mater.* 2400763 (2024) doi:10.1002/aenm.202400763.

10. Gabardo, C. M. *et al.* Continuous Carbon Dioxide Electroreduction to Concentrated Multi-carbon Products Using a Membrane Electrode Assembly. *Joule* **3**, 2777–2791 (2019).
